# CCL2 promotes metastasis and epithelial–mesenchymal transition of non‐small cell lung cancer via PI3K/Akt/mTOR and autophagy pathways

**DOI:** 10.1111/cpr.13560

**Published:** 2023-10-18

**Authors:** Hui Xu, Jin Wang, Murad Al‐Nusaif, Huipeng Ma, Weidong Le

**Affiliations:** ^1^ Liaoning Provincial Key Laboratory for Research on the Pathogenic Mechanisms of Neurological Diseases The First Affiliated Hospital of Dalian Medical University Dalian China; ^2^ Department of Thoracic Surgery The First Affiliated Hospital of Dalian Medical University Dalian China; ^3^ College of Medical Laboratory Dalian Medical University Dalian China; ^4^ Institute of Neurology, Sichuan Academy of Medical Science‐Sichuan Provincial Hospital Medical School of UESTC Chengdu China

## Abstract

In non‐small cell lung cancer (NSCLC), metastasis is the most common phenotype, and autophagy plays a vital role in its regulation. However, there are limited data on how autophagy‐related genes and metastasis‐related genes affect NSCLC progression. Our goal was to identify the genes that regulate autophagy and metastasis in NSCLC, and to assess the underlying mechanisms in this current study. RNA sequencing data from public databases were used to screen differentially expressed autophagy‐ and metastasis‐associated genes. Enrichment analyses and immune correlations were conducted to identify hub genes and potential regulating pathways in NSCLC. In this study, we found that CCL2 expression was highly expressed in NSCLC tissues and high CCL2 level was correlated with strong infiltration in lung tissues from NSCLC patients. Overexpression of CCL2 can enhance the metastasis of NSCLC cells in nude mice. Furthermore, CCL2 activated the PI3K/Akt/mTOR signalling pathway axis, promoted epithelial–mesenchymal transition (EMT), and blocked the autophagic flux in NSCLC cells. Therefore, our results indicate that CCL2 promotes metastasis and EMT of NSCLC via PI3K/Akt/mTOR axis and autophagy signalling pathways. We believe that CCL2 could be a probable target for the diagnosis and therapeutics of NSCLC, and this study may expand our understanding of lung cancer.

## Graphical abstract

Our research investigated the biological roles of CCL2 in NSCLC and discovered that it effectively enhances the migratory and invasive traits of NSCLC cells. Moreover, our findings indicate that CCL2 might promote EMT in NSCLC cells by modulating autophagy and the PI3K/Akt/mTOR signalling pathways.

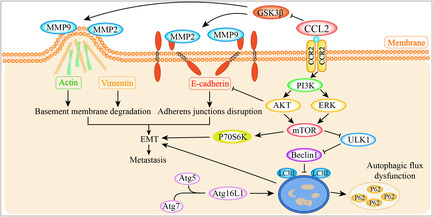

## 1 INTRODUCTION

There is no doubt that lung cancer is the most common form of cancer on a global scale, and it is the leading cause of cancer‐related mortality.^1^ Most lung cancers are non‐small cell lung cancers (NSCLCs), which constitute about 85% of lung cancer cases.^2^ For patients with lung cancer, chemotherapy remains a necessary adjuvant treatment, regardless of their chosen therapeutic option. However, chemotherapy drugs have significant adverse effects, and their effectiveness is not always satisfactory.^3^ Thus, identifying and characterising novel oncogenes attributed to NSCLC progression may help identify novel targets for the therapy of NSCLC.

Metastasis is the most significant contributor to cancer deaths, accounting for more than 90% of cancer‐related deaths.^4^ Among all tumours, lung cancer is the most serious regarding metastatic and mortality rates.^5^, ^6^, ^7^ In recent decades, many studies have investigated the factors contributing to tumour metastasis.^8^, ^9^, ^10^ According to the results, tumour metastases are greatly influenced by epithelial–mesenchymal transitions (EMT).^11^, ^12^ EMT is a dynamic and reversible process that allows polarised epithelial cells to be transited to mesenchymal cells. Several studies have shown that EMT‐inducing cytokines lead to disruption of adhesions between cell–cell and cell‐extracellular matrix (ECM), then endowing cancer cells with metastatic capability.^13^ Besides, growing evidence suggests that autophagy is vital in tumour metastasis.^14^, ^15^ It has been demonstrated that breast cancer metastasis is inhibited due to the autophagy‐induced degrading of NBR1.^16^ Metastasis of hepatocellular carcinoma was found to be hampered by SOCS5 because of its autophagy‐suppressing effect.^17^ Furthermore, several studies have shown that the PI3K/Akt and mTOR signalling pathways regulate autophagy.^18^, ^19^ However, there are still few reports about EMT and autophagy in NSCLC. Therefore, identifying genes involved in metastasis‐autophagy may provide NSCLC with potential therapeutic targets.

In this work, we sought to identify the differentially expressed genes associated with autophagy and metastasis (DEAMGs) and investigate the underlying regulating pathways in NSCLC. We thoroughly used publicly available data from Gene Expression Omnibus (GEO), Human Autophagy Database and Human Cancer Metastasis Database to screen DEAMGs. Protein–protein interactions (PPI), gene ontology (GO) and immune correlation analysis were performed to screen hub genes, and Kyoto Encyclopedia of Genes and Genomes (KEGG) enrichment analysis was performed to identify signalling pathways of DEAMGs. We found that CCL2 was associated with autophagy and metastasis and significantly correlated with immune cells.

After that, we analysed CCL2 expression in 12 NSCLC patients and found that CCL2 was particularly high expressed in high metastatic lung tissues. We also validated the expression of metastatic genes in lung cancer tissues and analysed the correlation between CCL2 and metastasis. Moreover, we investigated the effects of CCL2 on EMT and metastasis of NSCLC cells in vivo and in vitro. Here, we found that overexpression of CCL2 facilitated the metastasis of NSCLC cells in nude mice. Invasion experiments and wound‐healing migration studies in vitro showed that CCL2 significantly facilitated metastasis in NSCLC cells. Furthermore, after CCL2 inhibiting, the metastasis of the NSCLC cells was attenuated, suggesting the regulating effect of CCL2 on NSCLC. In addition, the expression of critical components of EMT, autophagy and PI3K‐Akt–mTOR pathways was examined via RT‐PCR, immunofluorescence staining and western blot assay. The decreased level of E‐cadherin and the increased level of vimentin, MMP‐2 and MMP‐9 proteins revealed the activation of EMT. Meanwhile, the increased expression of LC3II protein indicated autophagy initiation, whereas the decreased expression of Atg5, Atg7, Atg16L1, ULK1 and Beclin1 proteins and the increased expression of P62 protein suggested the inhibition of autophagic flux. The relative protein expression of LC3BII/LC3BI with and without CQ confirmed the impairment of autophagic flux in CCL2 overexpressed NSCLC cells. Finally, the relative expression of phosphorylated‐PI3K/total‐PI3K (p‐PI3K/t‐PI3K), phosphorylated‐Akt/total‐Akt (p‐Akt/t‐Akt), phosphorylated‐ERK/total‐ERK (p‐ERK/t‐ERK), phosphorylated‐P70S6K/total‐P70S6K (p‐P70S6K/t‐P70S6K), phosphorylated‐GSK3β/total‐GSK3β (p‐GSK3β/t‐GSK3β) and phosphorylated‐mTOR/total‐mTOR (p‐mTOR/t‐mTOR) further confirms the involvement of PI3K‐Akt–mTOR pathway in the development of NSCLC. By all accounts, we demonstrated that CCL2 promotes metastasis and EMT in NSCLC via PI3K/Akt/mTOR and autophagy pathways.

## 2 MATERIALS AND METHODS

### 2.1 Data preparation and quality control

The gene expression data of mRNA for NSCLC cells (GSE6013) was downloaded from GEO (https://www.ncbi.nlm.nih.gov/geo/). Datasets GSE6013 has a total of 27 samples, including five cases of NSCLC cells (A549 cells) and five cases of human bronchial epithelial cells (Beas 2B cells), based on platform GPL 570 ([HG‐U133_Plus_2] Affymetrix Human Genome U133 Plus 2.0 Array).^20^ Data quality was assessed and normalised with R software's ‘ggplot2’ package (version 3.6.3). After correcting the background, we controlled the gene expression matrix quality using principal component analysis (PCA).

### 2.2 Identification of DEGs

The DEGs were screened between A549 cells and Beas 2B cells using the GEO2R web tool in the GSE 6013 datasets. We calculated the log2FoldChange (logFC) and adjusted the *p*‐value (*P*. adj) using the default method to prevent false–positive data. To determine the significance of DEGs, |logFC| > 1 and *P*. adj <0.05 were set as cut‐off criteria. The autophagy‐related genes (AGs) were collected from the Human Autophagy Database (http://autophagy.lu), and the metastasis‐related genes (MGs) were collected from the Human Cancer Metastasis Database (http://hcmdb.i-sanger.com). Then, we identified DEAMGs using a Venn diagram package. The ‘ggplot2’ and ‘ComplexHeatmap’ packages were used to draw the Venn, volcano and complex heatmap.

### 2.3 Gene ontology and KEGG enrichment analysis

The functional annotation of gene ontology (GO) was carried out using the cluster profile (3.14.3) package in R software.^21^ The KEGG pathway enrichment analysis was carried out using the same package. The results with the *P*. adj <0.05 were considered noteworthy. The GO processes and KEGG‐enriched pathways were visualised using the GraphPad Prism software (9.0).

### 2.4 Construction of PPI networks and identification of hub genes

Based on the STRING database (https://cn.string-db.org/), the PPI networks were constructed and analysed using the Cytoscape software (3.9.1).^22^ According to the degree algorithm of the connected node, the top 10 hub DEAMGs were identified using the Cytohubba plugin.

### 2.5 NSCLC tissue sample collection and cell cultures

Twelve NSCLC tissue samples were surgically removed from NSCLC patients treated in the First Affiliated Hospital of Dalian Medical University. The use of these tissues was approved by the Ethics Committee of the First Affiliated Hospital of Dalian Medical University. All participants provided written informed consent.

The human BEAS‐2B bronchial epithelial cell line and A549 lung tumour cell line was obtained from the Cell Bank of the Chinese Academy of Sciences (Shanghai, China) and routinely cultured according to their protocols as recommended by the manufacturer. The cells were cultured in Dulbecco's Modified Eagle Medium (DMEM) essential medium (Life Technologies, Carlsbad, CA) supplemented with 10% foetal bovine serum (FBS, Gibco, USA) and 1% penicillin–streptomycin (PS, Beyotime, China) solution in a humidified incubator. The culture medium was refreshed every other day.

### 2.6 Plasmid transfection assay

We constructed CCL2 overexpressing cell models as previously described with minor modifications.^23^ Human CCL2 (pCMV‐SPORT6‐CCL2) and empty vector (pCMV‐SPORT6) plasmids were purchased from the MiaoLing Plasmid Platform (Wuhan, China). The plasmids were transfected into A549 cells with lipofectamine 6000 reagents (Beyotime, China) following the manufacturer's instructions. We screened for stably transfected A549 cells using a Geneticin medium (Thermo Fisher Scientific, USA).

### 2.7 Validation and immune correlation analysis of hub genes

Tissue expression of different hub DEAMGs was conducted based on the clinical NSCLC tissue samples. RT‐PCR data for samples from low metastatic and high metastatic tissues were used for analyses. Immune infiltration was analysed using the GSVA package and the ssGESA algorithm in R software.^24^ The corrections between 10 hub DEAMGs and 10 different types of immune cells (aDC, cytotoxic cells, DC, iDC, macrophages, NK cells, neutrophils, T cells, Th1 cells and TReg) were analysed using Spearman's correlation analysis.

### 2.8 Lung metastasis assay

Male BALB/c nude mice (5 weeks old) were obtained from Beijing VIEWSOLID Biotechnology Co., Ltd. Twenty four mice were divided into four groups randomly. Beas‐2B cells (control group), A549 cells (NSCLC group), A549 cells with empty vector plasmid (EV group) and CCL2‐stable overexpressed A549 cells (CCL2 group) were injected into the mouse tail (3 × 10^7^/mL, 0.1 mL, *n* = 6) for each group. Nude mice were fed for 6 weeks (weigh each mouse every 2 days) and then sacrificed. Lung tissues were removed, photographed, fixed and embedded in paraffin. Then, they were sectioned followed by haematoxylin and eosin (H&E) and IHC staining. The animal study was approved by the Institutional Animal Care and Use Committee of Dalian Medical University.

### 2.9 Design and fabrication of the microfluidic device

We designed a two‐layer microfluidic device with microchannels to stimulate lung micro vessels in vivo to study the invasion phenomenon of NSCLC cells. The microdevice comprised a poly‐dimethylsiloxane (PDMS) layer with micro vessel channels and a glass substrate layer. Each chip has four independent units connected by channels with a standard output port for waste. Four uniform regions in each unit represent the invasion of cells in vivo: one medium channel simulates the blood vessel and four gel channels allow the collagen to be infused with gelatin, which forms the extracellular matrix. This microdevice's vital characteristic was that the medium channels were almost three times higher than the collagen channels; hence, the liquid collagen gel infuses only in the gel channels due to the surface tension generated by the varied heights of the medium channels and gel channels.

The microfluidic device was fabricated using photolithography^25^ (Figure S1). The photoresist (SU8‐3035, MicroChem, USA) was repeated spin‐coated onto a clean glass until the thickness we needed, then patterned using traditional photolithography. To fabricate the gel channel structure, we coated SU‐83035 with UV light and exposed it once for a thickness of 70 μm. To simulate the microchannel structure for cell culture, the SU‐83035 was coated twice until the height was almost twice higher than the gel channels. Finally, the gel channels were about 70 μm tall, while the medium channels stood about 210 μm tall and 600 μm wide. The curing agent and PDMS base at a ratio of 1:10 by mass were mixed and poured into the mould, degassed in a vacuum and then baked at 80°C for 60 min. The concretionary PDMS gelatin was integrated peeled off from the mould, cut to an appropriate size and then irreversibly sealed to a clean glass following plasma treatment. Devices were UV sterilised before experiments.

### 2.10 Microfluidic chip invasion assay

Collagen type I solution (3 mg/mL, Corning, USA) was used to mimic the natural ECM in vivo. The solution was perfused into collagen channels and gelled at 37°C for 30 min. After that, the suspension of cells (5 × 10^4^ cells/mL) was gently injected into the medium channels of the microdevice. Afterward, the microdevices were flipped over for 5 min to assist the cells in adhering to the gel surface. After that, the chips were incubated in a humidified incubator, and the cells in microchannels were allowed to invade for 24 h.

### 2.11 Scratch wound healing assay

We used a scratch wound healing assay to determine the migration of tumour cells. Four groups of cells were cultured in 6‐well plates at 37°C for 48 h until 70%–80% confluence, and then we scratched the monolayer with a pipette tip (yellow, 200 μL) to generate scratch wounds. After that, the cells were incubated with DMEM essential medium (FBS free) at 37°C under normal conditions, and photographs were taken every 24 h to monitor cell migration.

### 2.12 Immunofluorescence staining and imaging

The samples were washed three times with PBS, fixed in paraformaldehyde (Sigma) for 30 min and then treated with 0.2% Triton‐X 100 solution (Sigma) for permeabilisation. Next, the samples were washed thrice and then non‐specific blocked for 45 min. Afterward, we incubated the samples at 4°C overnight with primary antibodies (Table S1). After washing with PBS thrice the next day, the samples were incubated with specific secondary antibodies (Table S1) for 1 h. Finally, the samples were washed and immersed in a PBS solution containing DAPI (Beyotime, China). Photographs were taken with a confocal microscope (Nikon A1R, Nikon, Japan) and processed with NIS‐Elements Viewer.

### 2.13 RNA isolation and quantitative realtime‐PCR

The total mRNA of lung tissue and cell samples was isolated using RNAiso Plus (Takara, Japan) and reverse‐transcribed to double‐strand cDNA using a PrimeScript RT reagent Kit with a gDNA eraser (TRAN, China). The relative gene expression was determined using a quantitative real‐time PCR assay with specific primers (Table S2). Gene expressions were quantified according to their relative abundance based on the 2^‐△△Ct^ method, and act‐1 was used as the normalised mRNA.

### 2.14 Western blot assay

Protein samples were separated using 6%–15% SDS‐PAGE and then electrophoretically transferred to PVDF membranes (0.2 μm /0.45 μm, Merck Millipore Ltd., Germany). The PVDF membranes were blocked in 5% evaporated milk for non‐specific sites binding for 1 h and then incubated with specific primary antibodies (Table S1) in PBS at 4°C overnight. The next day, the membranes were incubated with Horseradish Peroxidase (HRP)‐conjugated secondary antibodies (Table S1) for 1 h and then developed with an enhanced electrochemiluminescence (ECL) Plus procedure as specified by the manufacturer (Meilunbio, Dalian, China). Relative immunoblot signals were analysed with a FluorChem™ Q System (Protein Simple, USA). The density of GAPDH normalised the grey values.

### 2.15 Statistical analysis

We used GraphPad Prism 9.0 software for statistical analysis. All data were presented as the mean ± SD. Two‐tailed Student's *t*‐test was used to observe the differences between groups. We defined statistical significance as a *p* value less than 0.05. **** equals *p* < 0.0001; *** equals *p* < 0.001; ** equals *p* < 0.01; * equals *p* < 0.05; ns means no significance.

## 3 RESULTS

### 3.1 Identification and enrichment analysis of DEGs obtained from the GEO datasets

**FIGURE 1:**
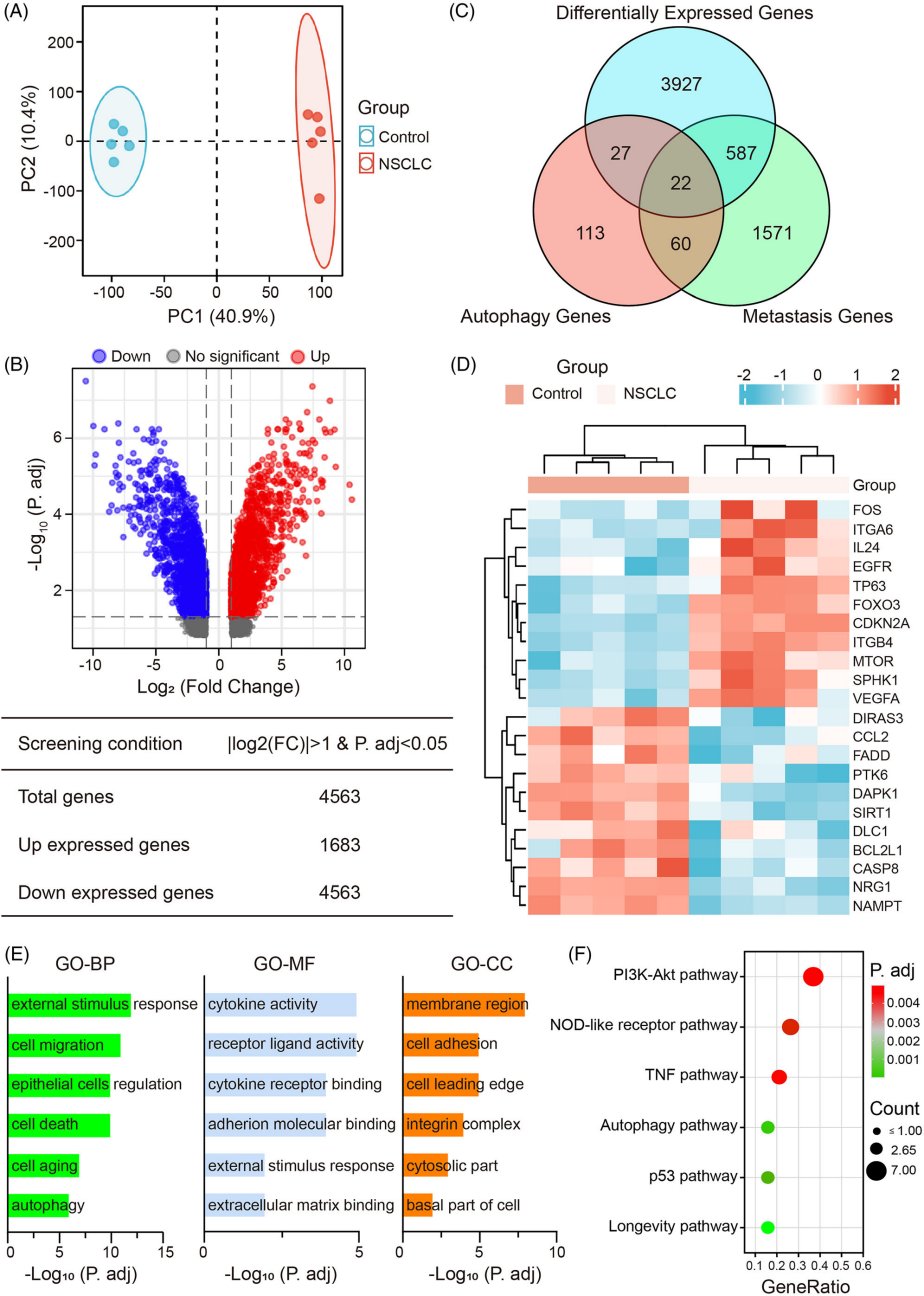
Integrated identification and enrichment analysis of DEAMGs obtained from the GEO datasets. (A) PCA analysis for related cell samples of the GSE 6013 dataset. The data between the control group and the NSCLC group are readily distinguished. (B) Volcano plot for DEGs screened from the GEO 6013 profile. Up‐regulated genes are represented by red dots (adj. *P* value <0.05 and log2FC ≥1); down‐regulated genes are represented by blue dots (adj. *P* value <0.05 and log2FC ≤ −1); black dots represent genes with no significant differences. (C) The Venn map of DEGs indicates the 22 intersectant DEAMGs of AGs, MGs and DEGs in the GSE6013 dataset. (D) Clustered heatmap of the 11 up‐regulated DEAMGs and 11 down‐regulated DEAMGs among control and NSCLC samples in GSE6013. (E) GO enrichment analysis of significant items in three functional groups (BP, CC and MF). (F) The bubble diagram of top six pathways distinguished by KEGG enrichment analysis between control and NSCLC cells. A bubble plot represents gene enrichment pathways, and the bubble size specifies the number of genes enriched in each pathway. AGs, autophagy‐related genes; DEAMGs, differentially expressed autophagy‐ and metastasis‐associated genes; MGs, metastasis‐related genes; NSCLC, non‐small cell lung cancer; PCA, principal component analysis.

The samples in the GSE 6013 dataset were first subjected to PCA, which revealed segregation based on sample type (Figure 1A). In addition, we found 4563 DEGs between control and NSCLC cells in this dataset, including 1683 up‐regulated genes and 1842 down‐regulated genes (Figure 1B). Then, we extracted 222 AGs and 2240 MGs from the Human Autophagy Database and the Human Cancer Metastasis Database, respectively. Twenty two DEAMGs were obtained by combining the genes from three databases (Figure 1C). The heatmap of 22 DEAMGs is presented in Figure 1D. According to the heatmap, 11 DEAMGs were up‐regulated, and 11 DEAMGs were down‐regulated (Table S3).

Functional enrichment analysis was carried out to investigate the gene ontology further and better visualise the differences in pathways of the 22 DEAMGs, including BP, CC, MF and KEGG (Figure 1E,F). Based on our findings, DEAMGs are significantly enriched in biological processes of cellular response to external stimulus, regulation of cell migration, epithelial cells, cell death, cell ageing and autophagy. Meanwhile, the DEAMGs were mainly enriched in membrane region, cell adhesion and cell leading edge by cellular component, and mainly enriched in receptor‐ligand activity, cytokine activity, cytokine receptor binding and cell adhesion molecular binding by molecular functions, respectively (Figure 1E). Moreover, enrichment and analysis of KEGG pathways were performed as well. The DEAMGs were significantly enriched in the NOD‐like receptor pathway, PI3K‐Akt pathway, TNF pathway, autophagy pathway, p53 pathway and longevity regulating pathway (Figure 1F). For instance, DEAMGs such as ITGB4, VEGFA, FOXO3, EGFR, BCL2L1, ITGA6 and MTOR were mainly implicated in the PI3K‐Akt pathway. DEAMGs such as FOS, VEGFA and CCL2 were associated with the autophagy signalling pathway. The DEAMGs enrichment results from the GO and KEGG enrichment analysis were summarised in Table S4.

### 3.2 Analysis of the PPI network and validation of DEAMGs

**FIGURE 2:**
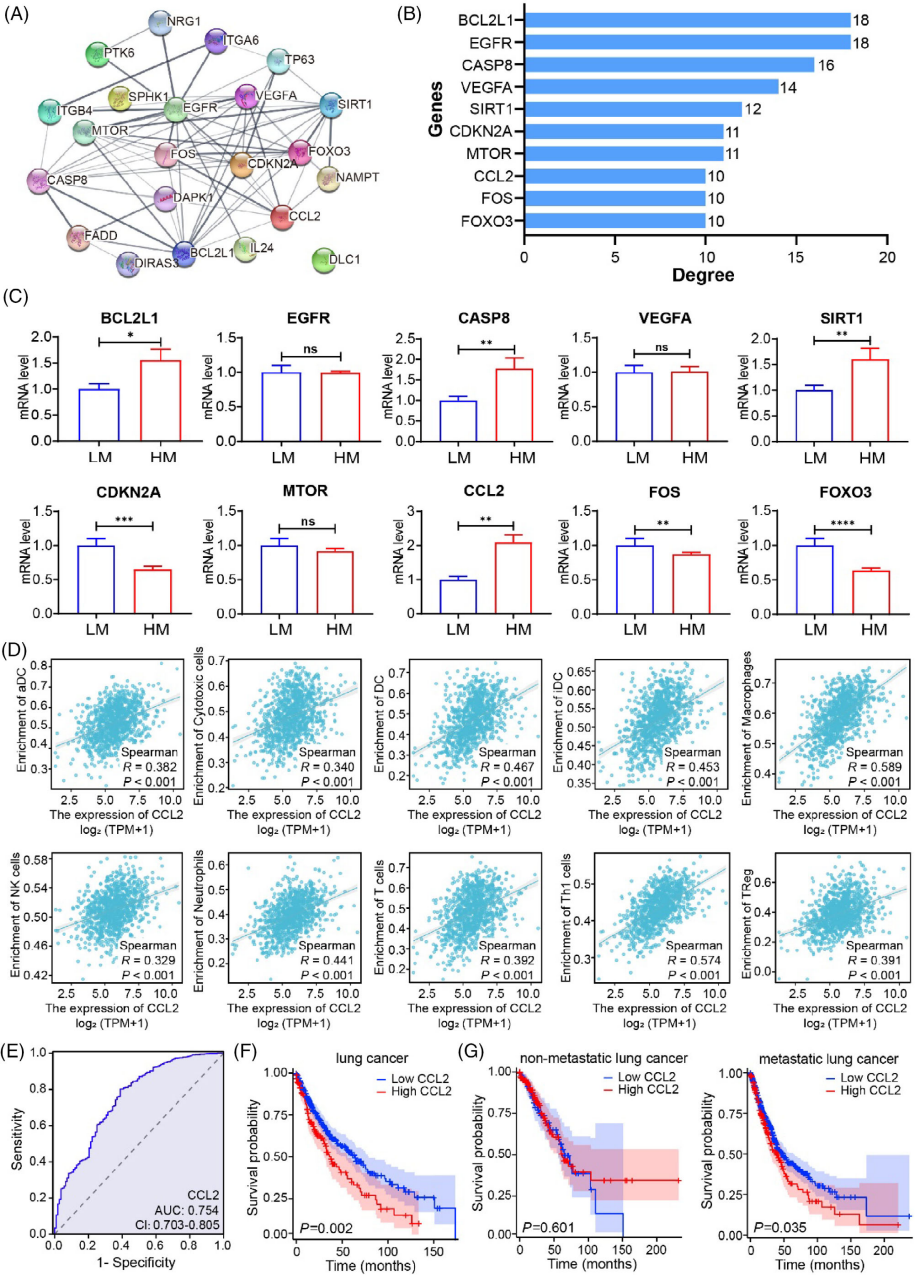
PPI network and hub DEAMGs identification of the DEGs. (A) PPI network of 22 DEAMGs. (B) Top 10 hub DEAMGs screened by degree score ranking. (C) The validation of hub DEAMGs in lung cancer tissues. (D) Correlation between hub DEAMGs and different immune cells in lung cancer. Significantly associated hub DEAMGs and immune cells were screened by |*R*| > 0.30 and *p* < 0.05. (E) ROC curve of CCL2 based on TCGA database. (F) Kaplan–Meier analysis of the relationship between CCL2 expression and overall survival in NSCLC patients. (G) Kaplan–Meier analysis of the relationship between CCL2 expression and overall survival in subgroup patients with or without metastasis. DEAMGs, differentially expressed autophagy‐ and metastasis‐associated genes; PPI, protein–protein interaction.

By combining the STRING database and Cytoscape software, we established a PPI network of DEAMGs to explore their interactions. According to Figure 2A, there are 22 nodes and 98 edges in the PPI network. A ranking of these DEAMGs was determined by the degree of connectivity they have in the PPI network, and the top 10 genes with the highest degrees in this network were BCL2L1, EGFR, CASP8, VEGFA, SIRT1, CDKN2A, MTOR, CCL2, FOS and FOXO3 (Figure 2B). To further confirm the reliability of the GSE6013 dataset, we determined the expression of 10 hub DEAMGs in lung cancer tissues with low metastasis (LM) and high metastasis (HM). We found that the expression of up‐regulated hub DEAMGs BCL2L1, CASP8, SIRT1 and CCL2 in lung cancer tissues with high metastasis was dramatically increased compared with lung cancer tissues with low metastasis. In contrast, the expression of down‐regulated hub DEAMGs CDKN2A, FOS and FOXO3 was decreased, consistent with that in UCSC XENA. There was no significant difference in EGFR, VEGFA and MTOR expression between these tissues (Figure 2C).

To assess the specific regulating effect of the hub DEAMGs on NSCLC, we analysed the correlation between 10 hub DEAMGs and 10 different types of immune cells in lung cancer and adjacent tissues. Significantly associated hub DEAMGs and immune cells were screened by |*R*| > 0.30 and *p* < 0.05. According to the results (Figure 2D; Figures S2–S4), CCL2 was positively associated with aDC (*R* = 0.382, *p* < 0.001), cytotoxic cells (*R* = 0.340, *p* < 0.001), DC (*R* = 0.467, *p* < 0.001), iDC (*R* = 0.453, *p* < 0.001), macrophages (*R* = 0.589, *p* < 0.001), neutrophils (*R* = 0.441, *p* < 0.001), NK cells (*R* = 0.329, *p* < 0.001), T cells (*R* = 392, *p* < 0.001), Th1 cells (*R* = 0.574, *p* < 0.001) and Treg (*R* = 0.391, *p* < 0.001; Figure 2D). Consequently, CCL2 can be used as a target‐regulating gene for NSCLC.

To explore the clinical significance of CCL2, we classified CCL2 expression into low expression group and high expression group of 677 cases of NSCLC tissues based on the TCGA database (https://portal.gdc.cancer.gov). CCL2 protein expression was not correlated with gender (*p* = 0.076) but was significantly correlated with age (*p* < 0.05), histology type (*p* < 0.005), tumour size (*p* < 0.01) and TNM stage (*p* < 0.05), as determined by chi‐squared test (Table S5). We evaluated the diagnostic value of CCL2 expression in differentiating NSCLC tissues by ROC analysis. The area under the curve (AUC) of CCL2 was 0.754 (Figure 2E), suggesting that CCL2 has high sensitivity for NSCLC diagnosis. We subsequently explored the relationship between CCL2 expression and overall survival using Kaplan–Meier (KM) analysis and log‐rank test. As shown in Figure 2F, NSCLC patients with high CCL2 expression had a significantly shorter overall survival than those with low CCL2 expression (*p* = 0.002). Furthermore, we stratified patients with metastatic factor and analysed the association of CCL2 expression with the overall survival of these patients. KM analysis showed that high CCL2 expression could also predict poor overall survival in the patient subgroup with metastasis (*p* = 0.035), but not in patients with non‐metastasis (*p* = 0.601, Figure 2G). Taken together, these findings suggest that CCL2 expression is associated with the poor prognosis of NSCLC patients, especially in patients with metastasis.

### 3.3 Overexpression of CCL2 promotes the metastasis of NSCLC cells in vivo

**FIGURE 3:**
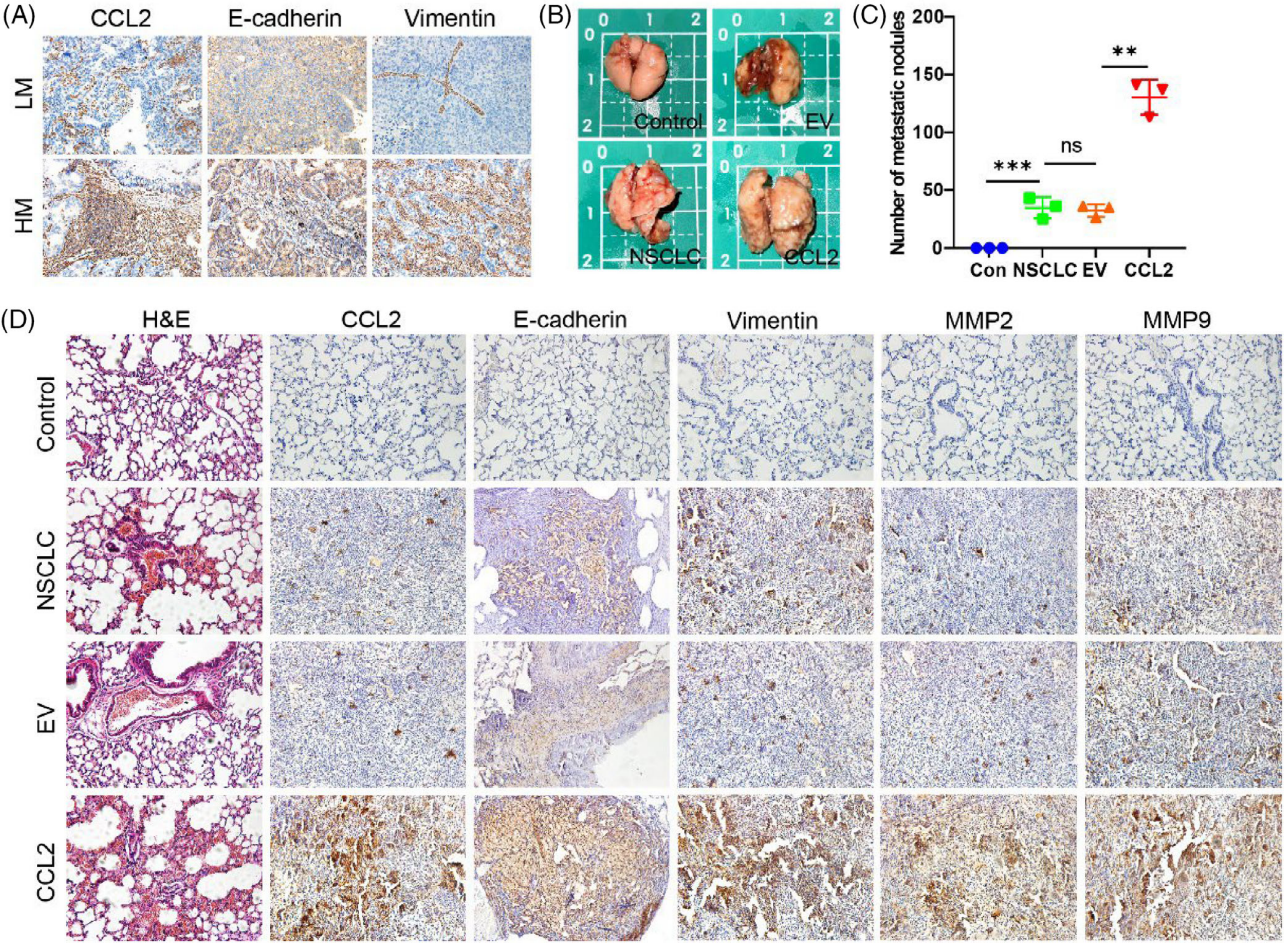
Overexpression of CCL2 promotes the metastasis of NSCLC cells in vivo. (A) CCL2, E‐cadherin and vimentin proteins were detected by IHC in the tissues of NSCLC patients with low metastasis and high metastasis. (B) Gross view of the lungs in the four groups of nude mice with or without metastatic nodules. (C) Statistical graph of lung metastatic nodules in the four groups of nude mice (*n* = 3, unpaired *t‐test*; **p < 0.01; ***p < 0.001). (D) H&E and IHC staining of nude mouse lungs. NSCLC, non‐small cell lung cancer.

To explore the correlation between CCL2 and metastasis, we detected the expression of CCL2 and metastatic proteins by IHC in the tissues of NSCLC patients with low metastasis and high metastasis. We found that CCL2 was highly expressed in the high metastatic lung cancer tissues but was less expressed in low metastatic lung cancer tissues. The expression of vimentin was higher, accompanied by significant degradation of E‐cadherin, resulting in diminished cell–cell adhesion in high metastatic lung cancer tissues (Figure 3A). The mice injected with CCL2‐overexpressed A549 cells developed more lung metastases than the mice inject with control A549 cells (Figure 3B,C). We detected the cell morphology by H&E staining and found that there were significantly more metastatic nodules in the CCL2 overexpressed lung tissues than that in NSCLC and EV groups (Figure 3D). We subsequently detected the EMT‐related markers in these lung tissues by IHC staining and found that overexpression of CCL2 regulated the expression of vimentin, MMP‐2 and MMP‐9. In addition, the overexpression of CCL2 promoted the degradation of E‐cadherin, leading to the disruption of intercellular tight junctions (Figure 3D). Collectively, these findings suggest that overexpression of CCL2 can accelerate the metastasis ability of NSCLC cells in vivo.

### 3.4 Design and fabrication of the microfluidic device

We fabricated a three‐dimensional microdevice reproducing the critical structural and functional characteristics of the lung tumour microenvironment (TME) in vitro (Figure S5A). The PDMS microdevice contains 16 independent units linked via microchannels (Figure S5B). Each unit has four uniform stromal gel regions and a medium channel for introducing fluidic flow and cells in chips (Figure S5C). The microdevice's compartmentalised channel configuration allows the fluidic flow to be manipulated and cells and nutrients to be delivered to channels independently. As a result of the parallel design of functional units, the invasion assay can be performed in a high throughput manner. The photo of the fabricated microdevice is shown in Figure S5D.

### 3.5 CCL2 promotes the metastasis of NSCLC cells

**FIGURE 4:**
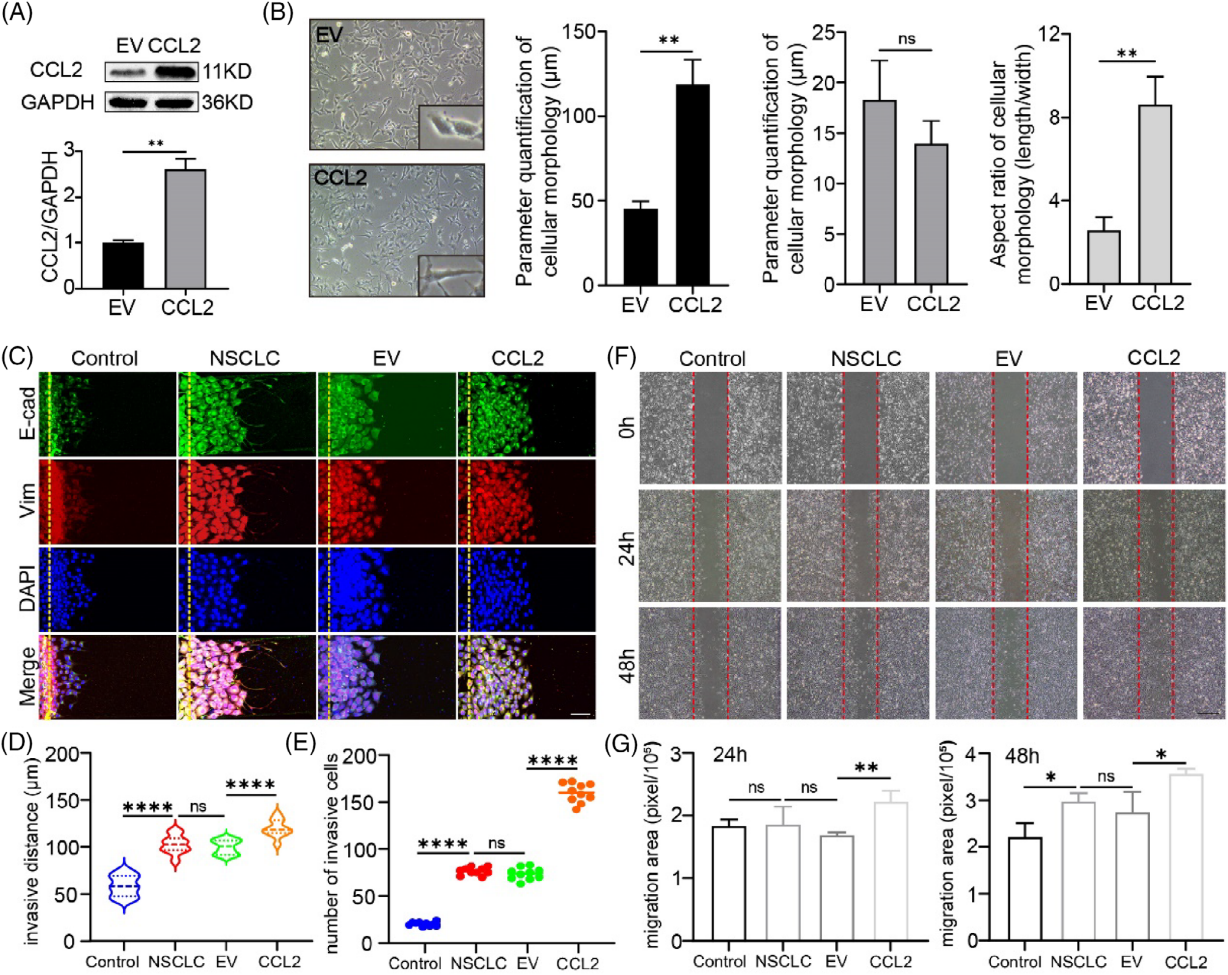
CCL2 promotes metastasis of NSCLC cells. (A) Efficacy of CCL2 overexpression in A549 cells. A549 cells transfected with empty plasmid were used as control (*n* = 3; **p < 0.01; unpaired *t‐test*). (B) Morphology of A549 cells overexpressed with CCL2 or control cells (*n* = 10; **p < 0.01; unpaired *t*‐test). (C) Invasion assay of NSCLC cells on microdevices for 24 h and immunofluorescent stains against E‐cadherin and vimentin (Scale bar = 50 μm). (D–E) Quantitation of distances and numbers of invasive cells. BEAS‐2B cells were used as control (*n* = 10; unpaired *t*‐test; ***p < 0.001; ****p < 0.0001). (F) Migration of NSCLC cells assessed by wound‐healing assays (Scale bar = 500 μm). (G) Quantitation of migration areas of wound‐healing assays (*n* = 3; unpaired *t*‐test; *p < 0.05; **p < 0.01; ***p < 0.001). NSCLC, non‐small cell lung cancer.

To confirm the transfection efficiency of control and CCL2 plasmids, we detect the protein expression of CCL2 in A549 cells. As shown in Figure 4A, the expression of CCL2 protein in transfected A549 cells increased compared to the controlled EV group. After that, we examined the regulation of CCL2 on the morphological changes of NSCLC cells. As assessed by a microscope, after transfected with CCL2 plasmid, we observed that the length of the NSCLC cells was significantly longer than in control cells. In contrast, the width of the NSCLC cells was not changed obviously, suggesting that CCL2 caused the polar changes in the cell morphology and promoted the mesenchymal transition of NSCLC cells (Figure 4B).

Mesenchymal transformation confers cells with enhanced invasive ability. To investigate whether CCL2 promotes a metastatic phenomenon of tumour cells, we evaluated the invasion of lung tumour cells using a microfluidic chip assay (Figure 4C–E). To establish the functional stroma barrier and invasion area, rat tail collagen type I was perfused into the collagen channels to mimic the stroma area. According to the scanning electron microscope (SEM) records, when the concentration of collagen type I was 3 mg/mL, the collagen gel was the densest and no apparent collagen filaments were found. Therefore, we used 3 mg/mL as the concentration of the extracellular matrix (Figure S6). As shown in Figure 4C, CCL2 significantly enhanced NSCLC cell invasion, including invasive distance and invasive cell numbers, and invasive cells exhibited weaker E‐cadherin and more robust vimentin expression than control cells. Pharmacologic CCL2 blocker further reduced cell invasion, indicating that the overexpression of CCL2 induced this metastasis (Figure S7A,B). Wound healing migration assays were performed to confirm this result and study the regulating effect of CCL2 on NSCLC cells. CCL2 increased the migratory ability of NSCLC cells in a time‐dependent manner (Figure 4F,G), while bindarit substantially inhibited CCL2's promoting development (Figure S7C,D). These findings indicate that CCL2 promotes the metastasis of NSCLC.

### 3.6 CCL2 regulated the expressions of EMT‐related genes and proteins in NSCLC cells

**FIGURE 5:**
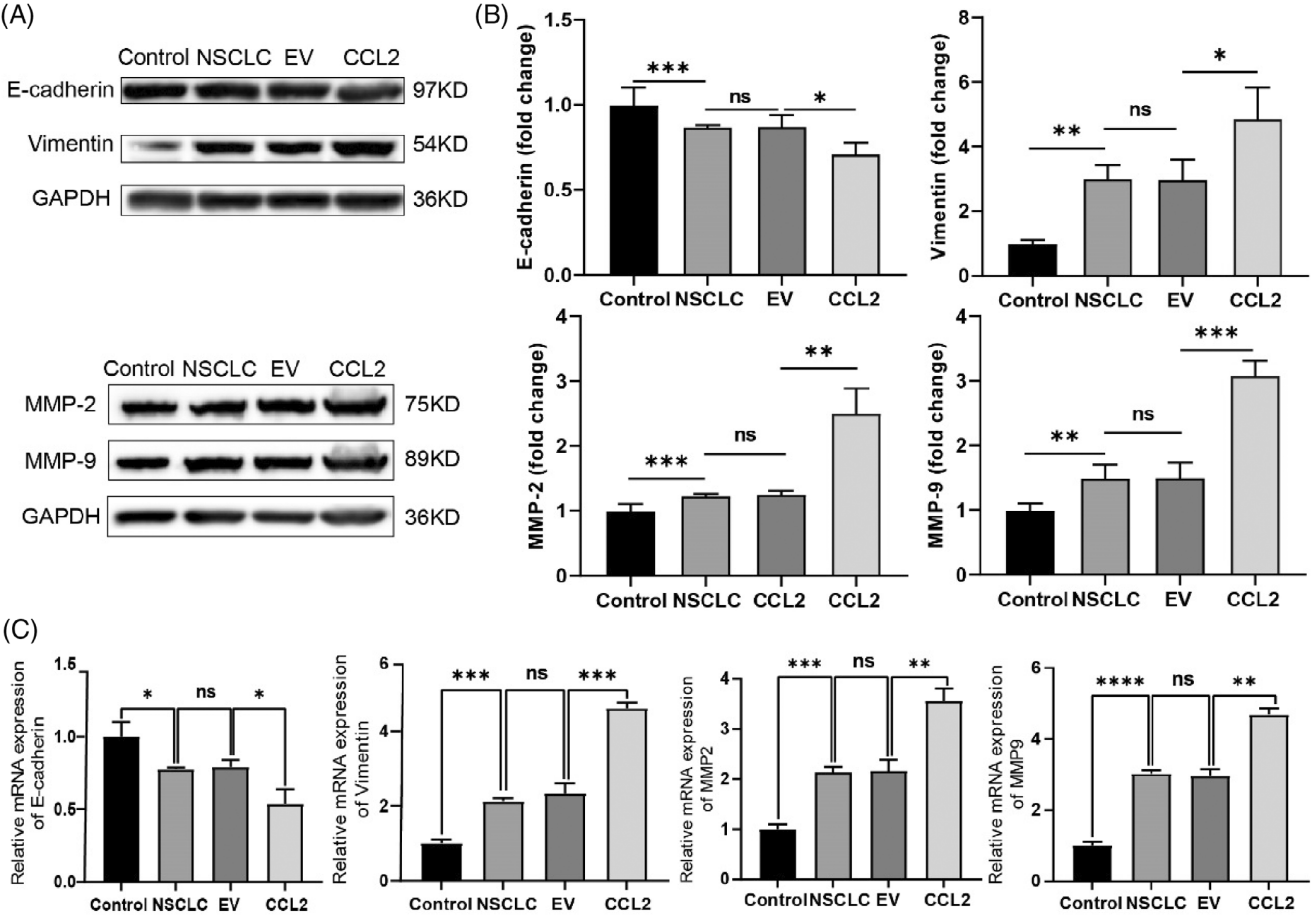
Expression and quantitation of the EMT proteins and genes in NSCLC cells. (A) Western blot validation of EMT regulating proteins in NSCLC cells under different conditions. (B) Quantitative analysis of the EMT proteins in NSCLC cells. BEAS‐2B cells were used as control, and the relative band intensity was normalised to GAPDH (*n* = 3, *p < 0.05, **p < 0.01, unpaired *t*‐test). (C) Quantitative analysis of RT‐PCR results of multiple EMT genes (*n* = 12; *p < 0.05; **p < 0.01; ***p < 0.001; ****p < 0.0001; ns, no significance; unpaired *t*‐test). EMT, epithelial–mesenchymal transition; NSCLC, non‐small cell lung cancer.

Specific EMT‐related genes can code mesenchymal transformation proteins and promote the metastasis phenomenon of tumour cells.^26^ Immunofluorescence staining and western blot assay were performed to assess the relative expression of EMT‐related proteins in NSCLC cells regulated by CCL2. The mRNA levels of EMT‐regulating genes were also calculated using RT‐PCR technology (Figure 5; Figure S8). Vimentin is a critical mesenchymal protein in metastatic cells. Under abnormal conditions, vimentin expression was positively associated with the metastatic degree of cells.^27^ It has been demonstrated that E‐cadherin, the first member of the cadherin superfamily, is essential for regulating cell–cell adhesion. The down‐regulation of E‐cadherin is correlated with invasive states in human cancers.^28^ During the EMT process, extracellular matrix (ECM) degradation occurs by releasing different matrix metalloproteases (MMPs). Among the MMPs, MMP‐2 and MMP‐9 play a vital role in the cleavage of fibronectin.^29^ According to Figure 5A,B and Figure S8A–C, a high level of E‐cadherin expression was detected, whereas the expression of vimentin, MMP‐2, and MMP‐9 was low in control cells. Compared with control cells, NSCLC cells expressed less E‐cadherin and more vimentin, MMP‐2 and MMP‐9.

Meanwhile, we found that CCL2 overexpression down‐regulated the E‐cadherin protein level and up‐regulated the expression level of vimentin, MMP‐2 and MMP‐9 significantly. Protein expression mirrors gene expression. We then examined the CCL2 knock‐in on the expression of EMT genes in vitro. As shown in Figure 5C and Figure S8D, the expression level of *E‐cadherin* and *ZO‐1* was decreased, and that of *vimentin*, *MMP‐2*, *MMP‐9*, *TWIST*, *SLUG*, *SNAIL*, *ZEB1* and *N‐cadherin* was significantly increased in CCL2 overexpressed NSCLC cells. Similarly, changes of EMT‐related genes and proteins in NSCLC cells can be inhibited by Bindarit (Figure S9). Based on these findings, we suggest that CCL2 promotes the expression of genes and proteins associated with EMT. Furthermore, CCL2's EMT‐promoting effect endows NSCLC cells with enhanced metastatic capabilities, implying that CCL2 enhances NSCLC invasion and migration.

### 3.7 CCL2 triggered autophagic flux dysfunction in NSCLC cells

**FIGURE 6:**
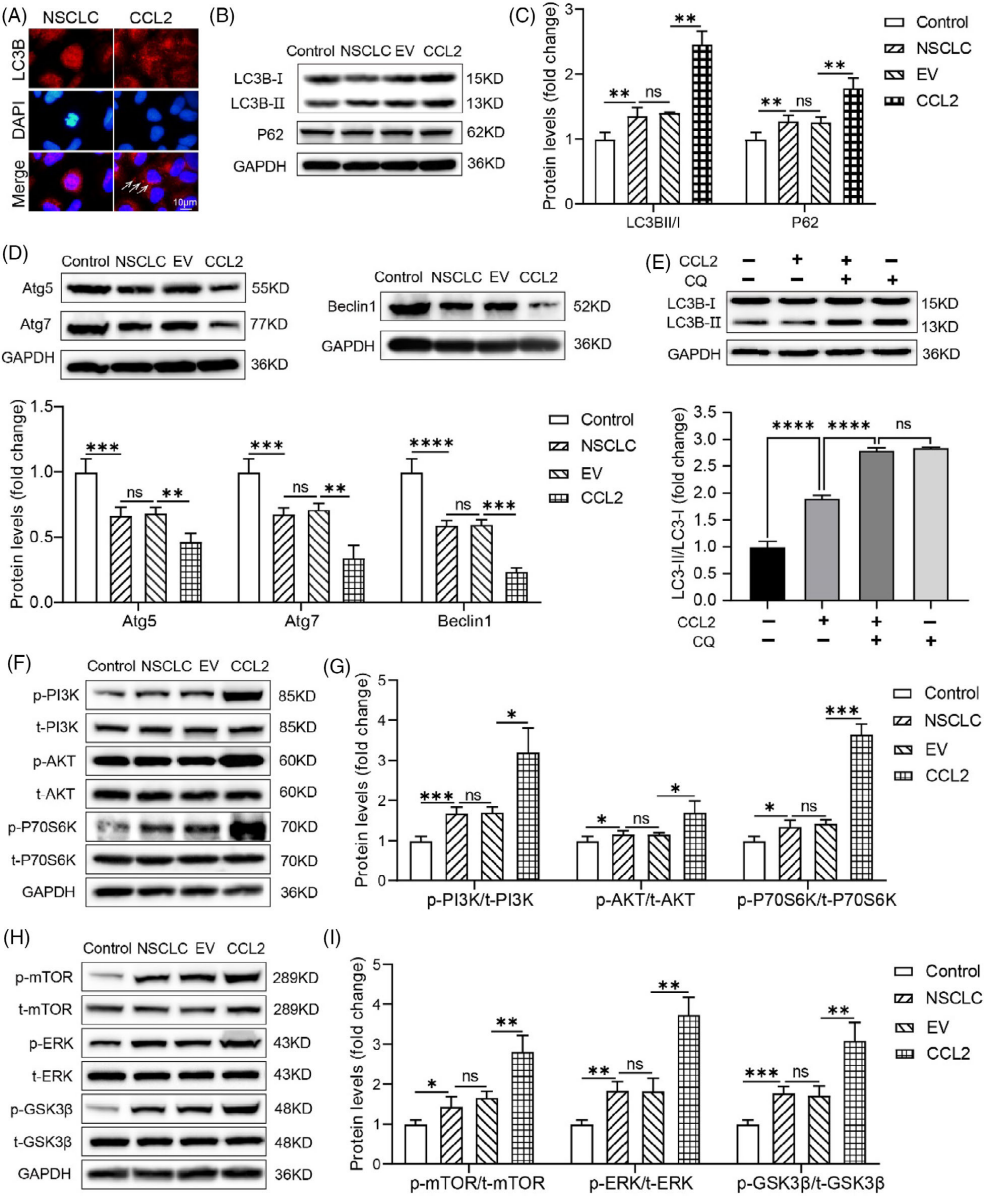
CCL2 triggered autophagic flux dysfunction and activated the PI3K/AKT/mTOR pathway in NSCLC cells. (A) Immunofluorescent staining for LC3B in CCL2 transfected A549 cells. LC3B puncta were pointed out with white arrows. Scale bar = 10 μm. (B–D) Western blot detection and quantitative analysis of expression levels of LC3B, P62, Atg5, Atg7 and Beclin1 proteins in CCL2 overexpressed A549 cells. BEAS‐2B cells were used as control, and the relative band intensity was normalised to GAPDH (*n* = 3; *p < 0.05; **p < 0.01; ***p < 0.001; ****p < 0.0001; unpaired *t*‐test). (E) Western blot detection and quantitative analysis of LC3B expression in the turnover assay. The relative band intensity was normalised to GAPDH (*n* = 3; ****p < 0.0001; ns, no significance; unpaired *t*‐test). (F–I) Western blot detection and quantitative analysis of phosphorylated/total PI3K, phosphorylated/total Akt, phosphorylated/total P70S6K, phosphorylated/total ERK, phosphorylated/total GSK3β, and phosphorylated/total mTOR proteins in CCL2 overexpressed A549 cells. BEAS‐2B cells were used as control, and the relative band intensity was normalised to GAPDH (*n* = 3; **p < 0.01; ***p < 0.001; unpaired *t*‐test). NSCLC, non‐small cell lung cancer.

The autophagic process is crucial for maintaining intracellular homeostasis. As a typical autophagy marker, LC3 usually disperses in the cytoplasm as LC3I. Under abnormal conditions, the autophagy process is activated, and cytoplasmic LC3I is cleaved into autophagosome‐associated LC3II, forming aggregation dots. Besides the typical transformation of LC3, many other distinct biomarkers participate in autophagy regulation. To better understand the autophagy‐modulating effect of CCL2 in A549 cells, the expression of LC3B, P62, Atg5, Atg7, Atg16L1, ULK1 and Beclin1 proteins was detected. The immunofluorescence staining results of LC3B showed that the CCL2 overexpressed A549 cells exhibited a significant increase of LC3B of the punctuate structure compared with untransfected A549 cells, indicating a conversion from the cytoplasmic (LC3I) to autophagosome‐associated (LC3II) form (Figure 6A). The western blot assay confirmed that LC3BI was successfully converted to LC3BII (Figure 6B,C). Our findings provided direct evidence that CCL2 promotes the conversion and accumulation of autophagosomes of A549 cells. We then tested the level of P62 protein and observed increased expression in CCL2 overexpressed A549 cells compared with control cells and untransfected A549 cells (Figure 6B,C), suggesting a blocked degradation of P62 in CCL2 transfected A549 cells. Likewise, the decreased expression of Atg5, Atg7, Beclin1, ULK1 and Atg16L1 proteins implied the disorder of autophagy in CCL2 overexpressed A549 cells (Figure 6D; Figure S10). These results indicated that CCL2 led to an autophagic flux impairment in NSCLC cells. It has been suggested that LC3BII levels may increase due to either activation of autophagy or a blockage of autophagic flux,^30^ so we effectuated an LC3BII turnover assay further to confirm the actual situation in CCL2 overexpressed A549 cells. We found that CQ (10 μM) did not further increase the level of LC3BII in the CCL2 overexpressed A549 cells compared with its administration alone (Figure 6E), indicating that the promoting effect of CCL2 on LC3BII accumulation may be due to impaired autophagic flux, rather than to an increased autophagy rate.

### 3.8 CCL2 activated the PI3K‐AKT–mTOR axis

The STRING database predicted the interaction between CCL2 and MTOR (Figure 2A). In addition, the KEGG enrichment results suggested that the PI3K/Akt signalling pathway may have a role in regulating the metastasis of NSCLC cells (Figure 1F). We assessed the protein levels of phospho‐PI3K, total‐PI3K, phospho‐Akt, total‐Akt, phospho‐P70S6K, total‐P70S6K, phospho‐ERK, total‐ERK, phospho‐GSK3β, total‐GSK3β, phospho‐mTOR and total‐mTOR in CCL2 overexpressed A549 cells to determine the probable pathways that CCL2 may regulate in NSCSC cells. We noticed that the amount of total PI3K was not changed obviously, whereas the amount of phosphorylated PI3K increased significantly. Consequently, compared to untreated A549 cells, the relative expression of p‐PI3K/t‐PI3K was increased dramatically in the CCL2 transfected A549 cells (Figure 6F,G), indicating that the PI3K was activated by phosphorylation. Moreover, in CCL2 overexpressed NSCLC cells, similar trends of p‐Akt/t‐Akt, p‐P70S6K/t‐P70S6K, p‐ERK/t‐ERK, p‐GSK3β/t‐GSK3β and p‐mTOR/t‐mTOR were observed (Figure 6F–I). Furthermore, we found that pharmacologic CCL2 blockade suppressed the phosphorylation effect of these proteins, indicating that CCL2 exerts a regulatory role on these pathways (Figure S11). These findings suggest that CCL2 may promote NSCLC cell metastasis and EMT via activating the PI3K/AKT/mTOR pathways.

## 4 DISCUSSION

More than 80,000 cancer deaths occur yearly due to NSCLC, and most patients with NSCLC have associated tumour metastasis.^31^ Autophagy is a dynamic and complex multiple‐step process and numerous signalling pathways are involved in autophagy. There is evidence that autophagy may be necessary for regulating metastasis in NSCLC.^32^, ^33^ Consequently, the prognostic prediction of NSCLC may be significantly improved by examining autophagy and metastasis‐related genes in patients. This study identified 22 DEAMGs associated with autophagy and metastasis in NSCLC based on gene expression profiles derived from the GSE 6013 dataset. According to the GO enrichment analysis, DEAMGs were significantly enriched in the cellular response to an external stimulus by biological processes, the membrane region by cellular component, and mainly in cytokine activity by molecular functions. Additionally, the KEGG analysis revealed that the DEAMGs were predominantly enriched in the NOD‐like receptor pathway, PI3K‐Akt pathway, TNF pathway, autophagy pathway, p53 pathway and longevity regulating pathway. The immune correlation results indicated that CCL2 positively correlated with immune cells, which can be used as a target‐regulating gene for NSCLC.

Although identifying potential DEAMGs associated with NSCLC can be carried out using bioinformatics, further experimental research is still necessary to confirm these findings. An earlier study demonstrated that CCL2 could activate the PI3k/Akt signalling in spinal cord injuries.^34^ The binding of CCL2 to CCR2 activates PI3 Kinases, which in turn activate Akt.^35^ Meanwhile, the CCL2/CCR2 axis activation can induce EMT by facilitating the recruitment of MMPs in the extracellular matrix, which causes the degradation of tight‐junction proteins and basement membrane.^36^, ^37^ We found that in NSCLC cells overexpressing CCL2, vimentin, MMP‐2 and MMP‐9 proteins were increased, while E‐cadherin expression was decreased. The detective results of EMT‐related genes and proteins indicated the attribution of high CCL2 levels in NSCLC cells.

**FIGURE 7:**
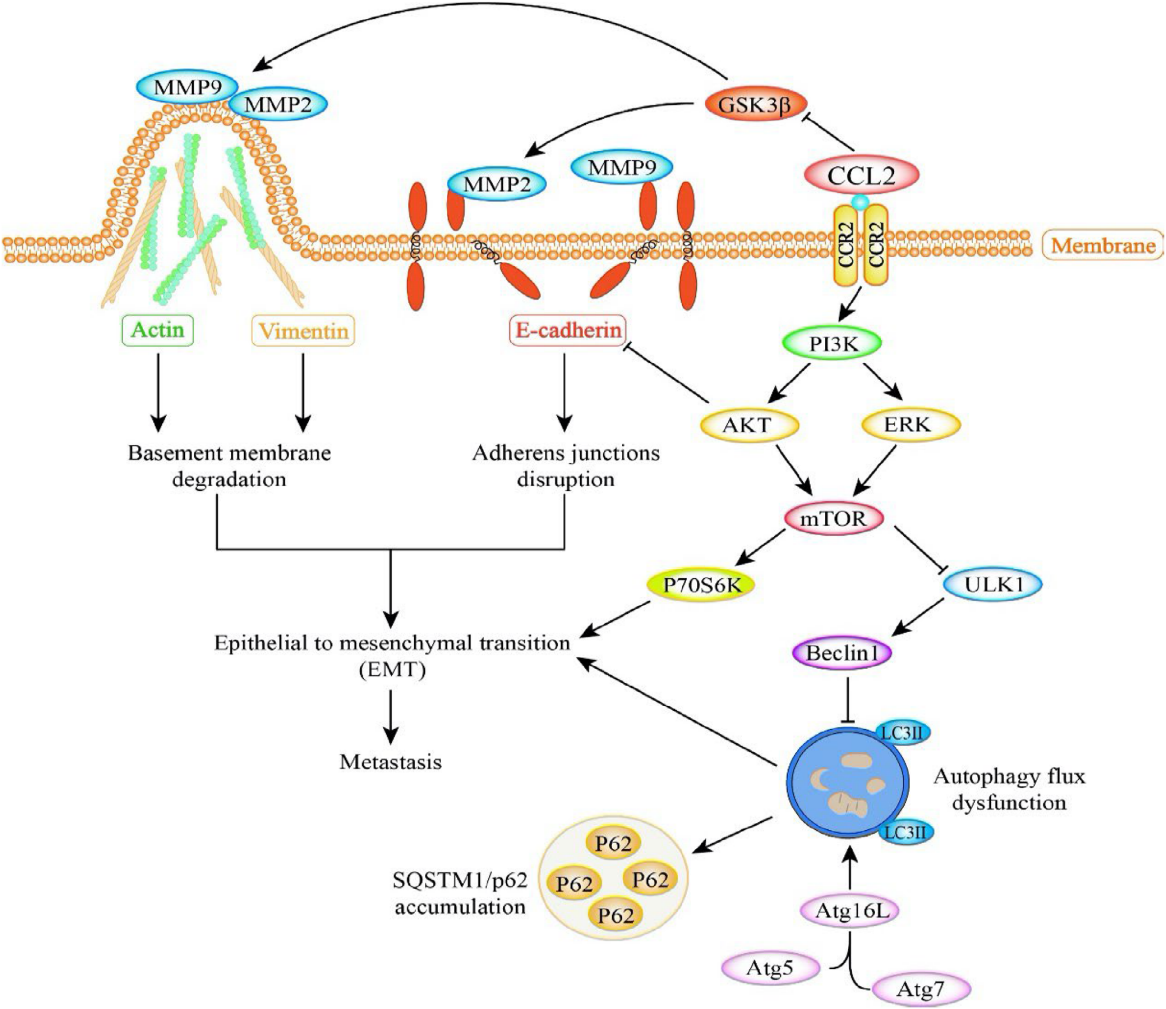
Schematic of CCL2 modulates metastasis and EMT in NSCLC cells via PI3K/Akt/mTOR and autophagy pathways. CCL2 down‐regulates the E‐cadherin protein level and up‐regulates the expression of vimentin, MMP‐2 and MMP‐9, indicating EMT activation. The relative expression level of autophagy proteins, especially LC3II, indicates the blocking effect of CCL2 on the autophagic flux. The activated mTOR and the impaired autophagic flux subsequently promote the activation of EMT, which results in metastasis in NSCLC cells. EMT, epithelial–mesenchymal transition; NSCLC, non‐small cell lung cancer.

It has been reported that autophagy is activated in the tumour microenvironment, and tumour promotion occurs when autophagy is lacking in tumour cells.^38^ In the current study, we found that CCL2 caused increased expression of LC3II protein in CCL2 overexpressed NSCLC cells, indicating autophagy initiation. However, the increased expression of the P62 protein showed abnormal degradation, suggesting the impairment of autophagic flux induced by CCL2. The decreased expression of Atg5, Atg7, ULK1, Beclin1 and Atg16L1 proteins also suggested abnormal autophagy. The result of the LC3BII turnover assay further confirms the blocking effect of CCL2 on autophagy. In addition, previous studies revealed that inhibition of autophagy and activation of mTOR specifically promoted EMT.^39^, ^40^ Therefore, the up‐regulated protein level of phosphorylated pI3K, Akt, P70S6K, ERK, GSK3β and mTOR, and blocked autophagic flux induced by CCL2 further enhance EMT activation, leading to metastasis of NSCLC cells. The possible modulating effect of CCL2 on metastasis and EMT and underlying autophagy and PI3K/Akt/mTOR pathway in NSCLC were summarised in Figure 7. Considering these results, we suggested that CCL2 may be a promising therapeutic target for human NSCLC.

## AUTHOR CONTRIBUTIONS

Hui Xu and Weidong Le initiated the concept. Hui Xu performed most of the bioinformatic analysis and experiments. Jin Wang collected surgically removed lung tissues from NSCLC patients. Huipeng Ma performed some western blot and RT‐PCR experiments and analysed the data. Hui Xu, Murad Al‐Nusaif and Weidong Le wrote the paper.

## FUNDING INFORMATION

This work was supported by the National Natural Science Foundation of China (NSFC 31700853).

## CONFLICT OF INTEREST STATEMENT

The authors have declared that no competing interest exists.

## Supporting information


**DATA S1:** Supporting Information.

## Data Availability

All data associated with this study are present in the paper or the Supplementary Materials.
