# Supplementary material for: CCL2 promotes metastasis and epithelial–mesenchymal transition of non‐small cell lung cancer via PI3K/Akt/mTOR and autophagy pathways
Source: Cell Prolif. 2023 Oct 18;57(3):e13560. doi: 10.1111/cpr.13560 (PMC10905333; doi:10.1111/cpr.13560)
Supplement: Supplementary file 1 — DATA S1: Supporting Information. [file CPR-57-e13560-s001.docx]

CCL2 promotes metastasis and epithelial-mesenchymal transition of non-small cell lung cancer via PI3K/Akt/mTOR and autophagy pathways

Hui Xu ^1^, Jin Wang ^2^, Murad Al-Nusaif ^1^, Huipeng Ma ^3^, Weidong Le ^1, 4 *^

^1^ Liaoning Provincial Key Laboratory for Research on the Pathogenic Mechanisms of Neurological Diseases, The first affiliated hospital of Dalian Medical University, Dalian 116021, People’s Republic of China.

^2^ Department of Thoracic Surgery, The first affiliated hospital of Dalian Medical University, Dalian 116021, People’s Republic of China.

^3^ College of Medical Laboratory, Dalian Medical University, Dalian 116044, People’s Republic of China.

^4^ Institute of Neurology, Sichuan Academy of Medical Science-Sichuan Provincial Hospital, Medical School of UESTC, Chengdu, 611731, People’s Republic of China.

Corresponding authors:

Tel/Fax: 0086-411-88135850.

Email: [wdle@sibs.ac.cn](mailto:wdle@sibs.ac.cn)

**Supplementary materials:**


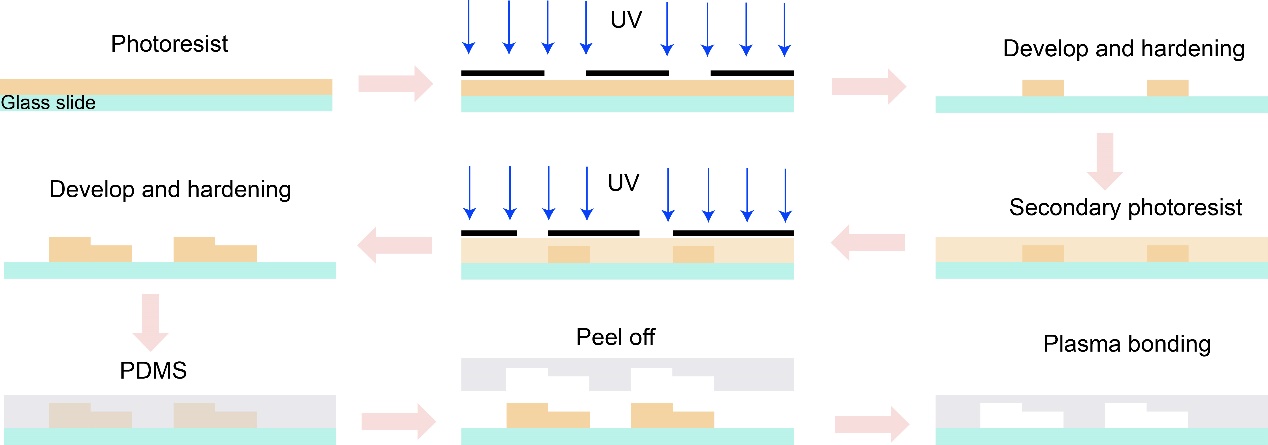


**Figure S1** The fabrication procedure of the microfluidic device.


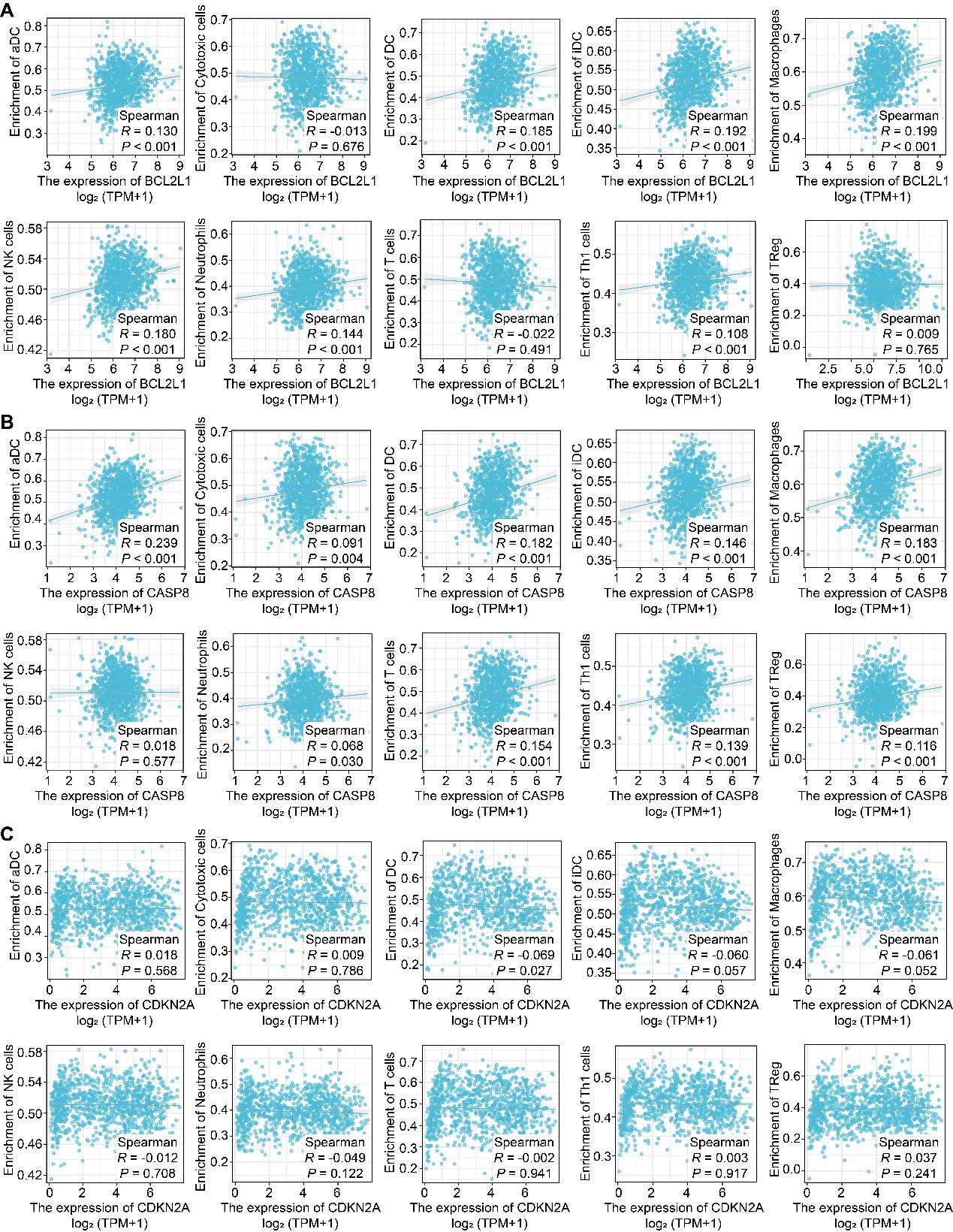


**Figure S2** Correlation between hub DEAMGs and different immune cells in lung cancer. (A) Immune correlations between BCL2L1 and immune cells in lung cancer. (B) Immune correlations between CASP8 and immune cells in lung cancer. (C) Immune correlations between CDKN2A and immune cells in lung cancer.


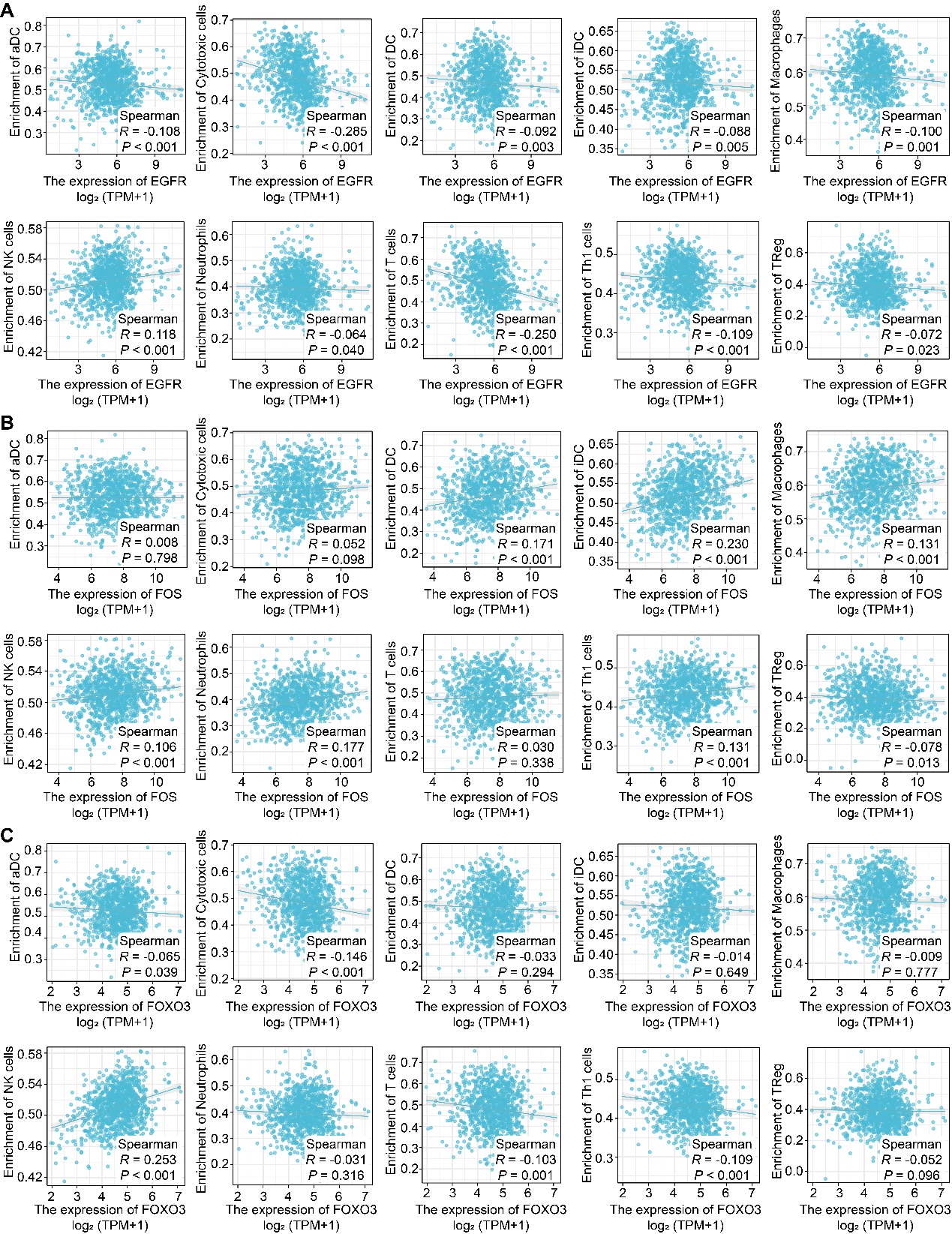


**Figure S3** Correlation between hub DEAMGs and different immune cells in lung cancer. (A) Immune correlations between EGFR and immune cells in lung cancer. (B) Immune correlations between FOS and immune cells in lung cancer. (C) Immune correlations between FOXO3 and immune cells in lung cancer.


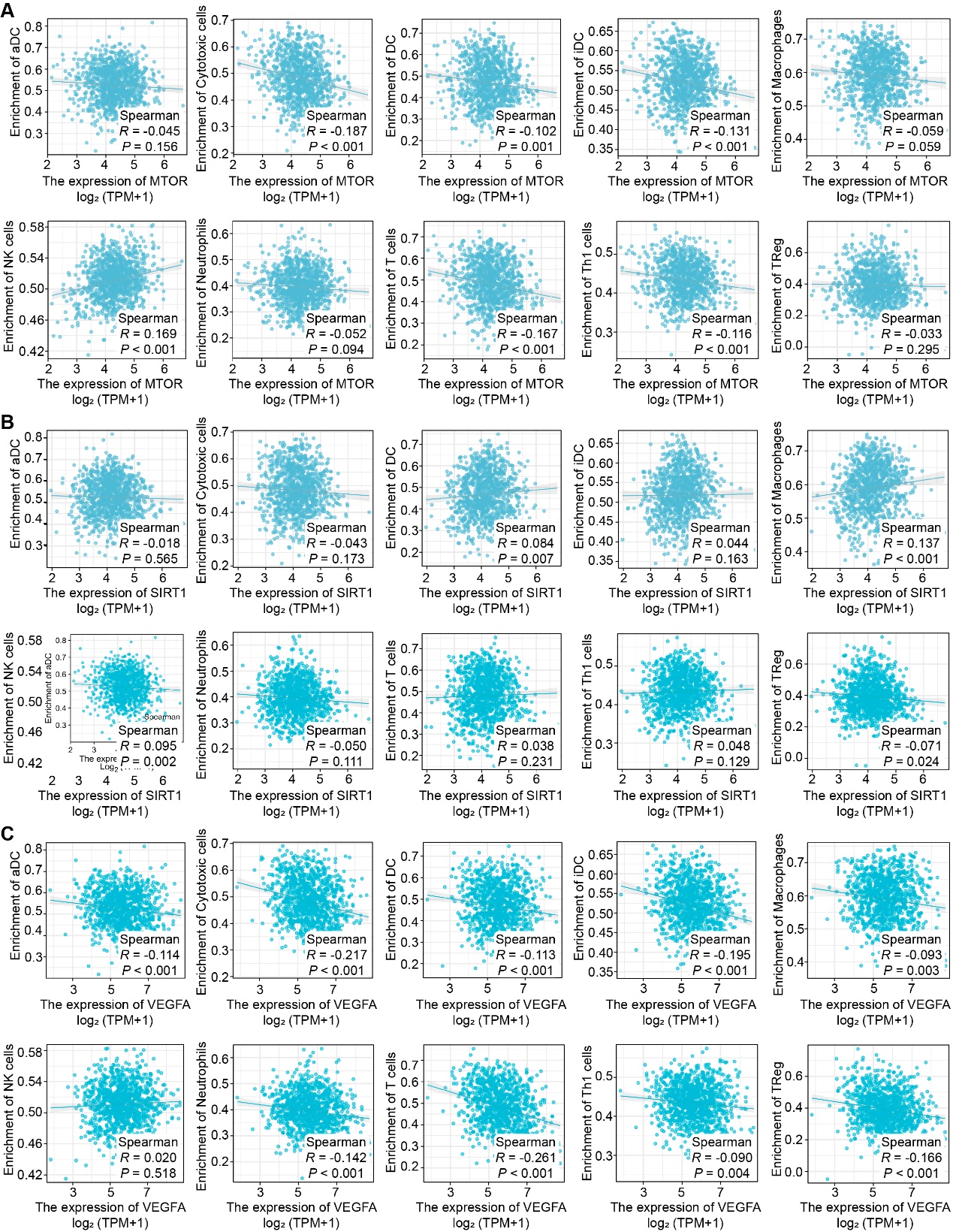


**Figure S4** Correlation between hub DEAMGs and different immune cells in lung cancer. (A) Immune correlations between MTOR and immune cells in lung cancer. (B) Immune correlations between SIRT1 and immune cells in lung cancer. (C) Immune correlations between VEGFA and immune cells in lung cancer.


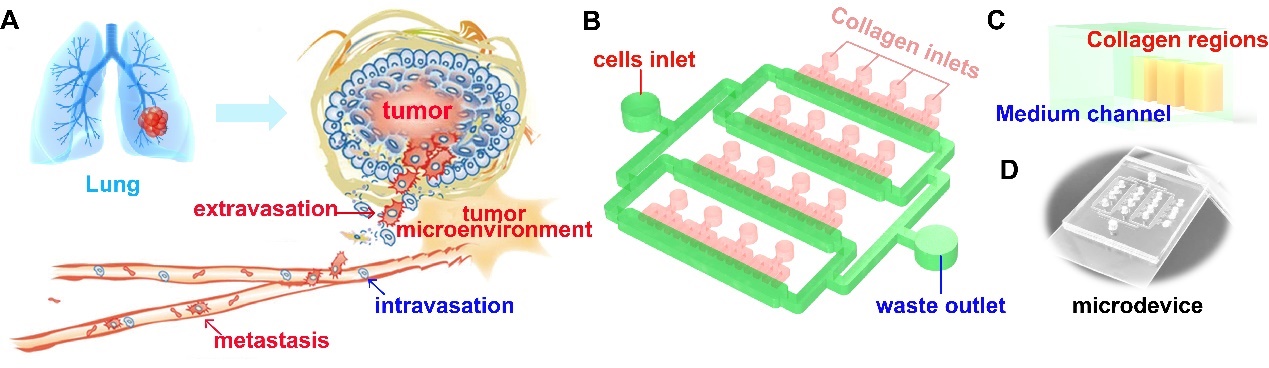


**Figure S5** Diagrammatic sketch of the microdevice for the invasion assay of lung tumor cells. (A) Schematic illustration of the TME and the procedure of tumor metastasis. (B) Chip design and structure of the microdevice. (C) Side view of the microdevice. (D) The photograph of the fabricated microdevice.


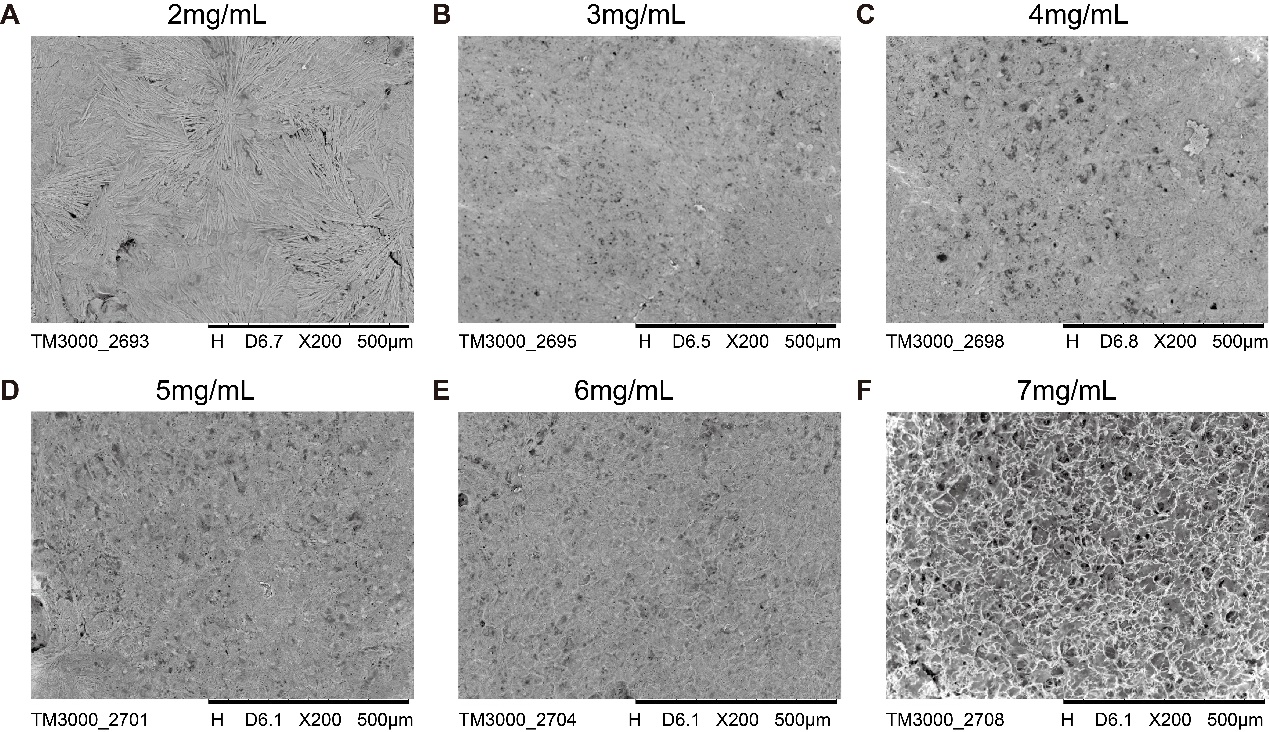


**Figure S6** Images of scanning electron microscope of collagen gels at different concentrations.


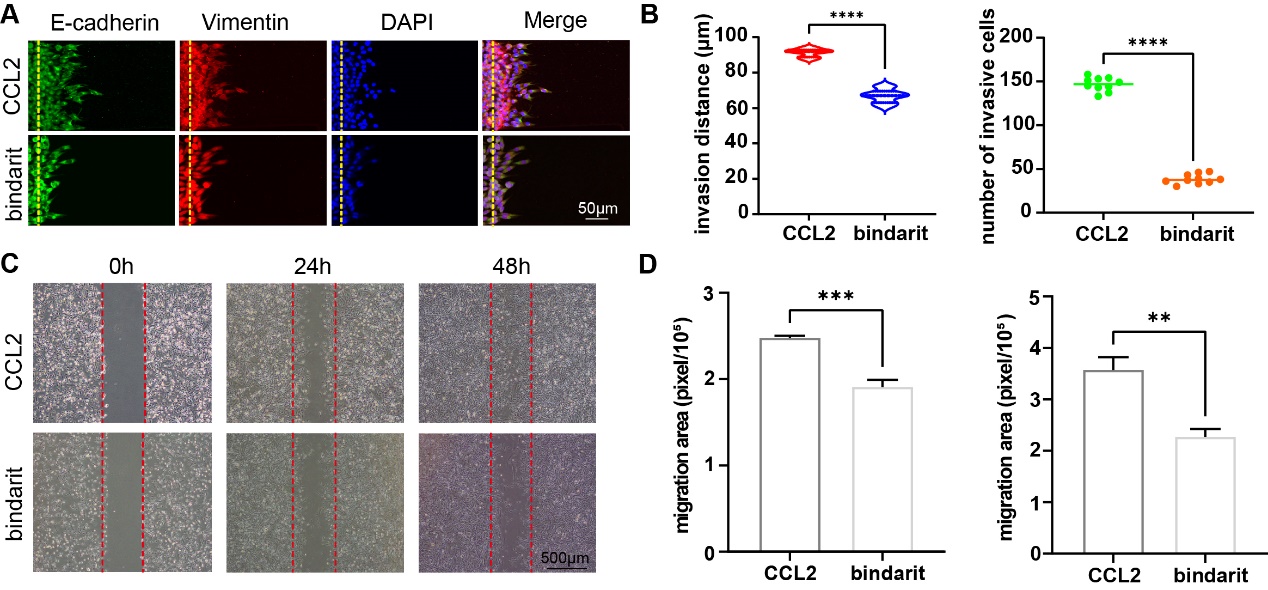


**Figure S7 Bindarit inhibited metastasis of NSCLC cells.** (A) Immunofluorescent stains against E-cadherin, vimentin, MMP-2, and MMP-9 proteins in NSCLC cells. (B) Quantitation of distances and numbers of invasive cells. (n=10; unpaired *t-test*; **** P < 0.0001). (C) Migration of NSCLC cells assessed by wound-healing assays. (Scale bar = 500 μm). (D) Quantitation of migration areas of wound-healing assays. (n=3; unpaired *t-test*; ** P < 0.01; *** P < 0.001).


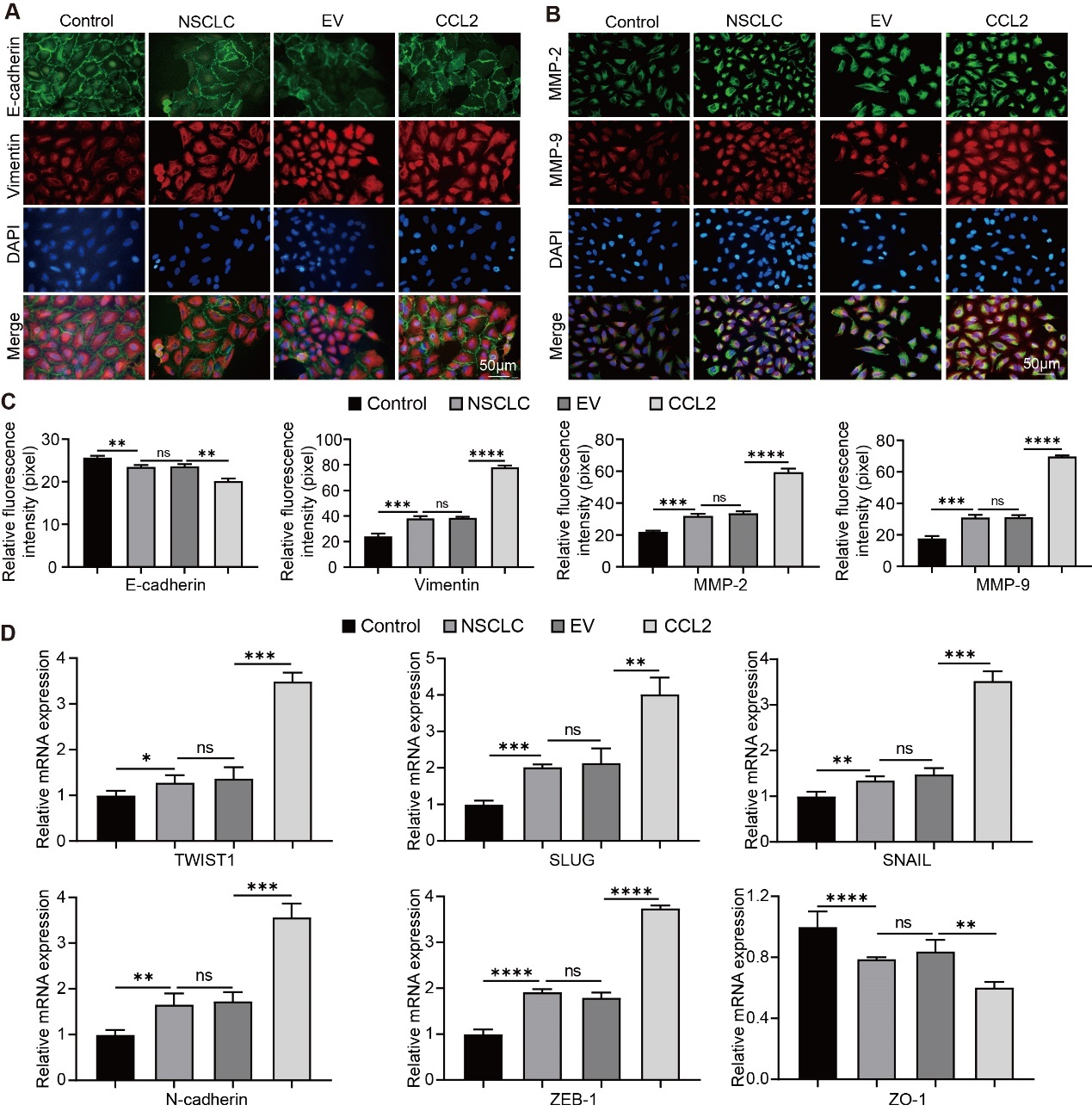


**Figure S8** **Relative expression and quantitation of the EMT proteins and genes in NSCLC cells.** (A-B) Immunofluorescent stains against E-cadherin, vimentin, MMP-2, and MMP-9 proteins in NSCLC cells. (C) Quantitative analysis of the EMT proteins in NSCLC cells. (n=3; * P < 0.05; ** P < 0.01; *** P < 0.001; **** P < 0.0001; unpaired *t-test*). (D) Quantitative analysis of RT-PCR results of multiple EMT genes in NSCLC cells. (n=9; * P < 0.05; ** P < 0.01; *** P < 0.001; **** P < 0.0001; unpaired *t-test*).


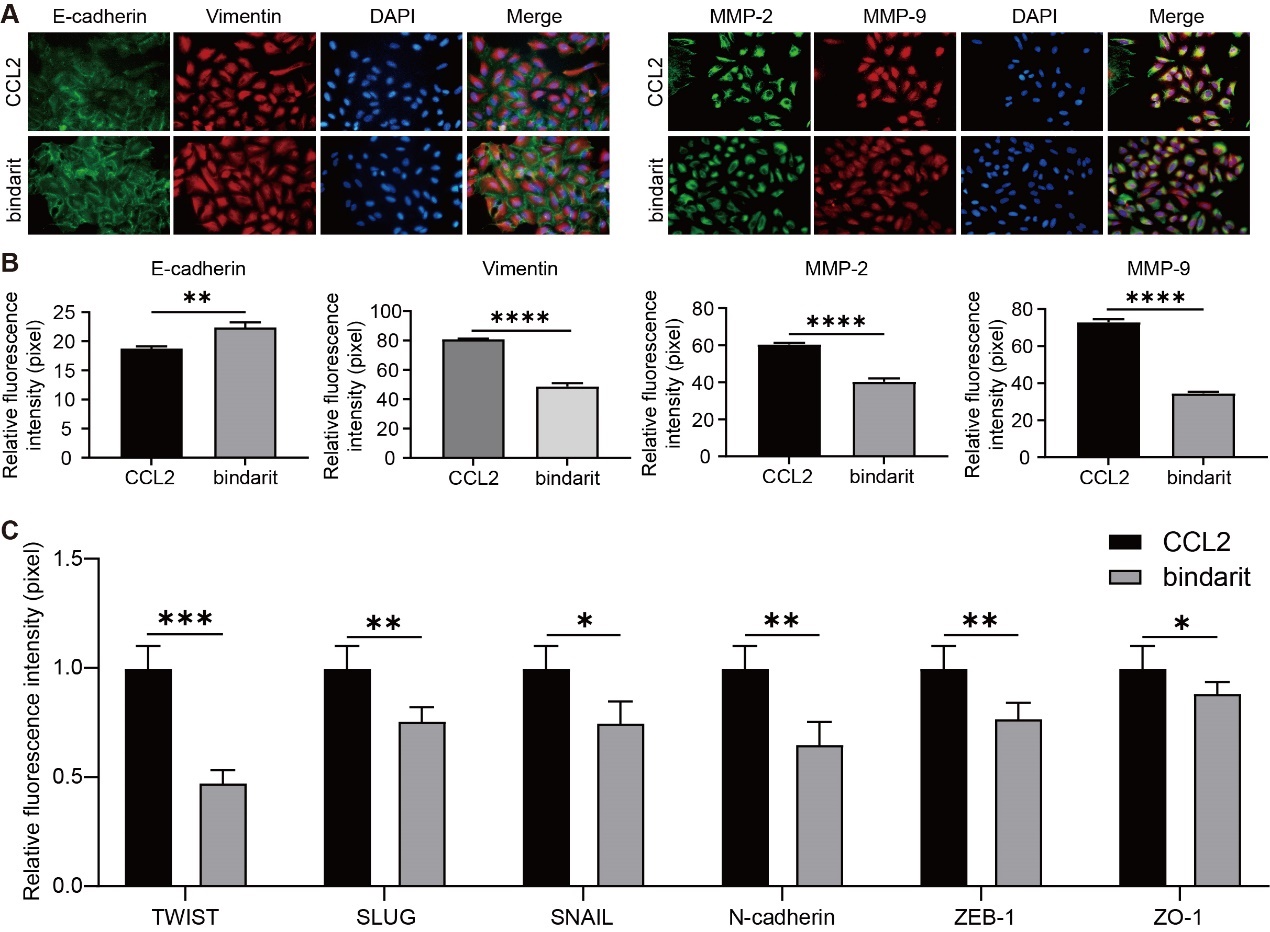


**Figure S9 Relative expression and quantitation of the EMT proteins and genes in NSCLC cells treated with bindarit.** (A) Immunofluorescent stains against E-cadherin, vimentin, MMP-2, and MMP-9 proteins in NSCLC cells. (B) Quantitative analysis of the EMT proteins in NSCLC cells. (n=3; ** P < 0.01; **** P < 0.0001; unpaired t-test). (C) Quantitative analysis of RT-PCR results of multiple EMT genes in NSCLC cells treated with bindarit. (n=9; * P < 0.05; ** P < 0.01; *** P < 0.001; unpaired t-test).


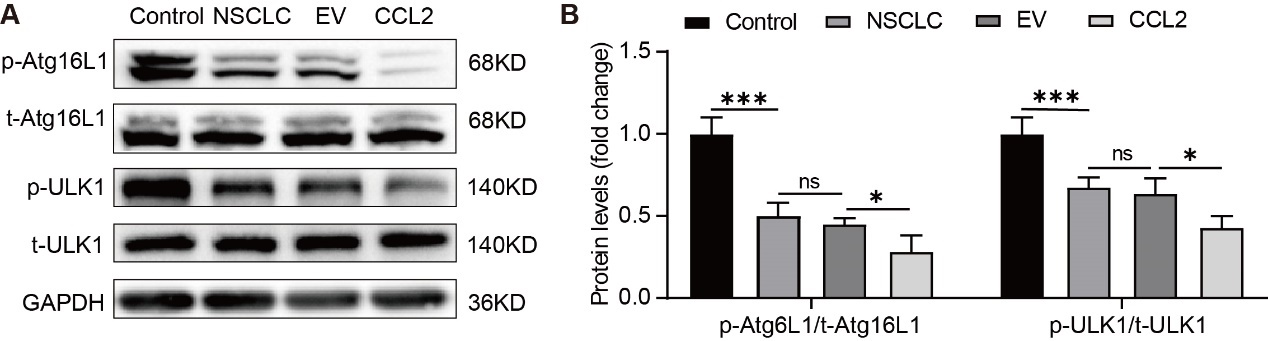


**Figure S10** Expression and quantitation of autophagy proteins in NSCLC cells. (A) Western blot validation of the Atg16L1 and ULK1 protein in NSCLC cells. (B) Quantitative analysis of the Atg16L1 and ULK1 protein in NSCLC cells. BEAS-2B cells were used as control, and the relative band intensity was normalized to GAPDH (n=3, * P < 0.05, *** P < 0.001, unpaired *t-test*).


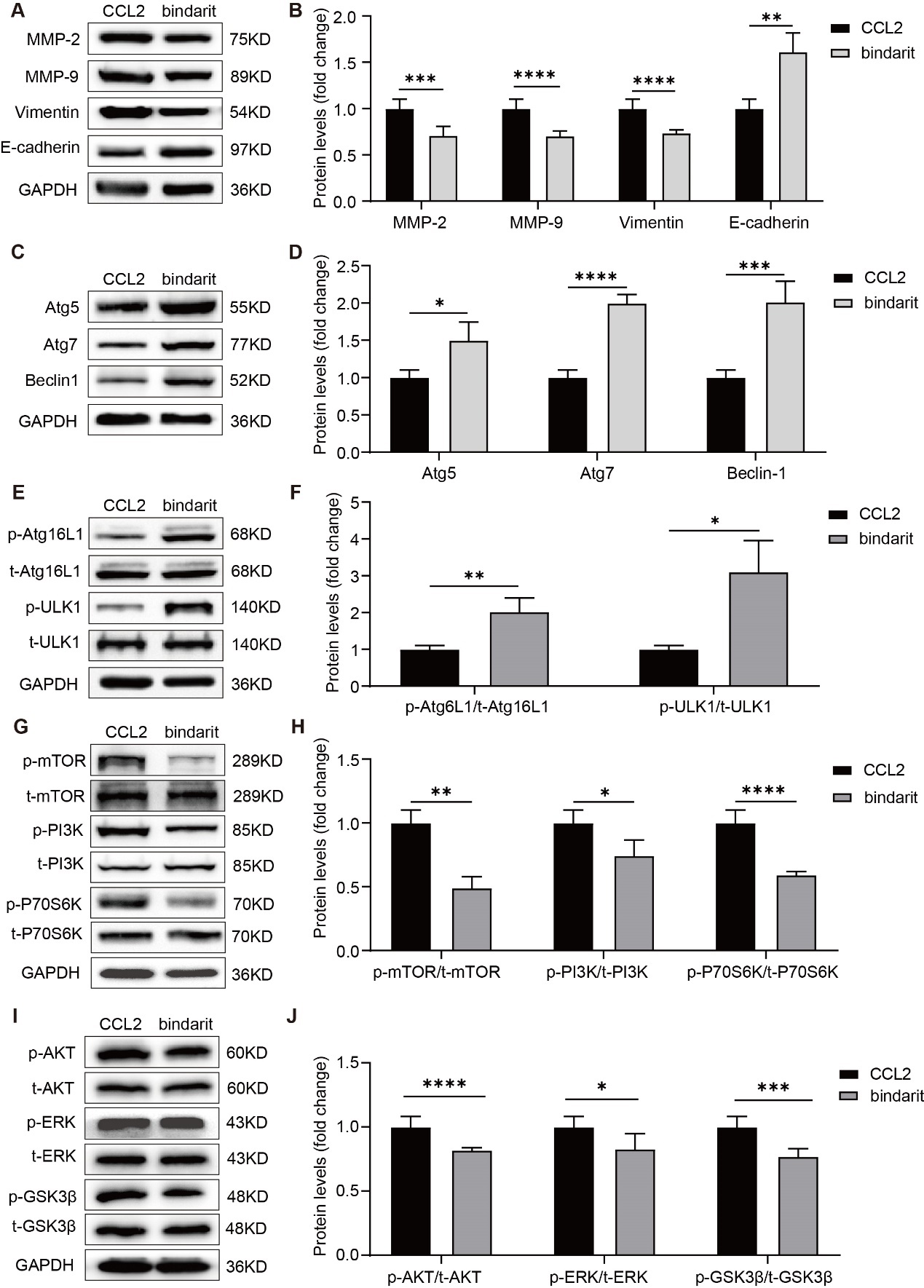


**Figure S11 Expression and quantitation of proteins in NSCLC cells treated with bindarit.** (A-B) Western blot validation and quantitative analysis of EMT-related proteins in NSCLC cells treated with bindarit. (C-F) Western blot validation and quantitative analysis of autophagy-related proteins in NSCLC cells treated with bindarit.

(G-J) Western blot validation and quantitative analysis of PI3K/Akt/mTOR axis-related proteins in NSCLC cells treated with bindarit. The relative band intensity was normalized to GAPDH (n=3, * P < 0.05, ** P < 0.01, *** P < 0.001, **** P < 0.0001, unpaired *t-test*).

Table S1 Antibodies used in this study.

| Names | Companies | Types | Application | Ratios |
| --- | --- | --- | --- | --- |
| Anti-Vimentin | Abcam | Primary antibody | IF/WB | 1:1000 |
| Anti-E-cadherin | ProteinTech | Primary antibody | IF/WB | 1:1000 |
| Anti-MMP2 | Abcam | Primary antibody | IF/WB | 1:1000 |
| Anti-MMP9 | Abcam | Primary antibody | IF/WB | 1:1000 |
| Anti-LC3B | Abcam | Primary antibody | IF/WB | 1:1000 |
| Anti-SQSTM1/p62 | Abcam | Primary antibody | WB | 1:2000 |
| Anti-CCL2 | ProteinTech | Primary antibody | WB | 1:2000 |
| Anti-Atg5L/Atg5 | Abcam | Primary antibody | WB | 1:2000 |
| Anti-Atg7 | Abcam | Primary antibody | WB | 1:2000 |
| Anti-Beclin1 | Abcam | Primary antibody | WB | 1:2000 |
| Anti-Atg16L1 | Abcam | Primary antibody | WB | 1:2000 |
| Anti-phospho-Atg16L1 | Abcam | Primary antibody | WB | 1:2000 |
| Anti-PI3 Kinase | CST | Primary antibody | WB | 1:2000 |
| Anti-phospho-PI3 Kinase | CST | Primary antibody | WB | 1:2000 |
| Anti-Akt | CST | Primary antibody | WB | 1:2000 |
| Anti-phospho- Akt | CST | Primary antibody | WB | 1:2000 |
| Anti-ERK | CST | Primary antibody | WB | 1:2000 |
| Anti-phospho- ERK | CST | Primary antibody | WB | 1:2000 |
| Anti-GSK3β | CST | Primary antibody | WB | 1:2000 |
| Anti-phospho-GSK3β | CST | Primary antibody | WB | 1:2000 |
| Anti-P70S6K | CST | Primary antibody | WB | 1:2000 |
| Anti-phospho-P70S6K | CST | Primary antibody | WB | 1:2000 |
| Anti-ULK1 | CST | Primary antibody | WB | 1:2000 |
| Anti-phospho-ULK1 | CST | Primary antibody | WB | 1:2000 |
| Anti-mTOR | CST | Primary antibody | WB | 1:2000 |
| Anti-phospho-mTOR | CST | Primary antibody | WB | 1:2000 |
| Anti-GAPDH | CST | Primary antibody | WB | 1:2000 |
| Anti-rabbit IgG (H+L) | CST | Secondary antibody | IF | 1:2000 |
| Anti-mouse IgG (H+L) | CST | Secondary antibody | IF | 1:2000 |
| HRP-conjugated Affinipure Goat Anti-Rabbit IgG(H+L) | ProteinTech | Secondary antibody | WB | 1:4000 |
| HRP-conjugated Affinipure Goat Anti-Mouse IgG(H+L) | ProteinTech | Secondary antibody | WB | 1:4000 |

Table S2 Primer sequences of RT-PCR used in this article.

| Primers | Forward (5’ to 3’) | Reverse (5’ to 3’) |
| --- | --- | --- |
| Vimentin | gccaggcaaagcaggagt | tgggtatcaaccagagggagt |
| E-cadherin | acgcattgccacatacac | accttccatgacagaccc |
| N-cadherin | atcctactggacggttcg | ttggctaatggcacttga |
| MMP-2 | ccgtcgcccatcatcaagtt | ctgtctggggcagtccaaag |
| MMP-9 | gggacgcagacatcgtcatc | tcgtcatcgtcgaaatgggc |
| ZO-1 | ggaaatgatgaggcagaa | ggtccgtatagcttgagg |
| TWIST1 | gtccgcagtcttacgaggag | ccagcttgagggtctgaatc |
| SLUG | caaggacacattagaactcacac | ctacacagcagccagattc |
| SNAIL | catccttctcactgccatgga | aggcagaggacacagaaccaga |
| ZEB1 | aagtggcggtagatggta | ttgtagcgactggatttt |
| ACT-1 | tagttgcgttacaccctttcttg | caccttcaccgttccagtttt |
| BCL2L1 | caacccatcctggcacct | gcatctccttgtctacgcttt |
| EGFR | catctccgaaagccaaca | cgacggtcctccaagtag |
| CASP8 | ggtaatgacaatctcggactc | aaaggtcgtggtcaaagc |
| VEGFA | ccttgctgctctacctcc | cctttcctcgaactgatttt |
| SIRT1 | tatttatgctcgccttgc | cctgttccagcgtgtcta |
| CDKN2A | ttcctggacacgctggtggt | ctatgcgggcatggttactgc |
| MTOR | gctgtcatccctttatcg | tcttcttcttctccctgtagtc |
| CCL2 | tgtgcctgctgctcatag | gtttgggtttgcttgtcc |
| FOS | gtctccagtgccaacttcat | cagccatcttattcctttcc |
| FOXO3 | acggctcactctgtccca | ttgccagttccctcattct |

Table S3 Data sources of ComplexHeatmap.

| Overlapped genes | Regulating effect | logFC | P value | adj. P value |
| --- | --- | --- | --- | --- |
| CCL2 | Upregulated | 5.44E+00 | 2.17E-04 | 3.11E-03 |
| NRG1 | Upregulated | 5.16E+00 | 5.53E-07 | 4.29E-05 |
| DAPK1 | Upregulated | 2.50E+00 | 1.29E-05 | 3.89E-04 |
| NAMPT | Upregulated | 2.40E+00 | 1.26E-06 | 7.37E-05 |
| DIRAS3 | Upregulated | 1.46E+00 | 1.24E-02 | 5.87E-02 |
| PTK6 | Upregulated | 1.30E+00 | 6.01E-03 | 3.48E-02 |
| DLC1 | Upregulated | 1.23E+00 | 1.44E-02 | 6.52E-02 |
| FADD | Upregulated | 1.21E+00 | 6.67E-03 | 3.75E-02 |
| BCL2L1 | Upregulated | 1.17E+00 | 6.71E-03 | 3.77E-02 |
| SIRT1 | Upregulated | 1.11E+00 | 4.26E-04 | 5.14E-03 |
| CASP8 | Upregulated | 1.08E+00 | 2.08E-03 | 1.64E-02 |
| CDKN2A | Downregulated | -5.03E+00 | 9.45E-09 | 3.59E-06 |
| ITGB4 | Downregulated | -3.56E+00 | 1.76E-07 | 1.95E-05 |
| IL24 | Downregulated | -3.23E+00 | 7.40E-04 | 7.69E-03 |
| SPHK1 | Downregulated | -2.73E+00 | 7.53E-05 | 1.41E-03 |
| TP63 | Downregulated | -2.56E+00 | 2.37E-04 | 3.32E-03 |
| FOS | Downregulated | -1.89E+00 | 2.25E-02 | 8.98E-02 |
| VEGFA | Downregulated | -1.71E+00 | 8.22E-04 | 8.29E-03 |
| FOXO3 | Downregulated | -1.37E+00 | 1.11E-04 | 1.91E-03 |
| EGFR | Downregulated | -1.26E+00 | 1.01E-02 | 5.06E-02 |
| ITGA6 | Downregulated | -1.06E+00 | 1.56E-02 | 6.91E-02 |
| MTOR | Downregulated | -1.06E+00 | 1.44E-03 | 1.25E-02 |

Table S4 DEAMGs in GO and KEGG enrichment analysis.

| Ontology | Description | GeneRatio | P. adj | Genes | Counts |
| --- | --- | --- | --- | --- | --- |
| BP | cellular response to external stimulus | 0.55 | 1.06E-02 | NAMPT/FOS/EGFR/FADD/SIRT1/CASP8/ITGA6/MTOR/FOXO3/CCL2/IL24/DAPK1 | 12 |
| BP | regulation of cell migration | 0.50 | 2.06E-02 | CCL2/NRG1/IL24/FOXO3/DLC1/VEGFA/SIRT1/MTOR/FADD/EGFR/ITGA6 | 11 |
| BP | regulation of epithelial cells | 0.45 | 1.64E-02 | TP63/VEGFA/EGFR/SIRT1/MTOR/CCL2/EGFR/FOXO3/BCL2L1/PTK6 | 10 |
| BP | regulation of cell death | 0.45 | 1.18E-02 | CCL2/TP63/NAMPT/FOS/FOXO3/FADD/BCL2L1/SIRT1/CASP8/MTOR | 10 |
| BP | regulation of cell aging | 0.32 | 6.89E-03 | CDKN2A/TP63/NAMPT/FOS/FOXO3/SIRT1/MTOR | 7 |
| BP | regulation of autophagy | 0.27 | 1.52E-02 | ITGB4/DAPK1/NAMPT/FOXO3/SIRT1/MTOR | 6 |
| CC | membrane region | 0.36 | 2.27E-04 | FOXO3/BCL2L1/CASP8/MTOR/EGFR/DLC1/FADD/SIRT1 | 8 |
| CC | cell adhesion | 0.23 | 2.22E-03 | ITGB4/EGFR/DLC1/ITGA6/VEGFA | 5 |
| CC | cell leading edge | 0.23 | 1.08E-02 | ITGB4/PTK6/DLC1/SPHK1/EGFR | 5 |
| CC | integrin complex | 0.18 | 1.15E-02 | ITGB4/ITGA6/MTOR/SIRT1 | 4 |
| CC | cytosolic part | 0.14 | 1.59E-02 | FADD/CASP8/MTOR | 3 |
| CC | basal part of cell | 0.09 | 8.74E-03 | EGFR/ITGA6 | 2 |
| MF | cytokine activity | 0.23 | 6.29E-06 | CCL2/NRG1/IL24/NAMPT/VEGFA | 5 |
| MF | receptor ligand activity | 0.23 | 2.63E-04 | CCL2/NRG1/IL24/NAMPT/VEGFA | 5 |
| MF | cytokine receptor binding | 0.18 | 3.88E-04 | CCL2/VEGFA/FADD/CASP8 | 4 |
| MF | cell adhesion molecule binding | 0.18 | 3.05E-03 | NRG1/ITGB4/EGFR/ITGA6 | 4 |
| MF | tumor necrosis factor receptor binding | 0.09 | 6.71E-04 | FADD/CASP8 | 2 |
| MF | extracellular matrix binding | 0.09 | 2.26E-03 | VEGFA/ITGA6 | 2 |
| KEGG | PI3K-Akt signaling pathway | 0.37 | 9.35E-06 | ITGB4/VEGFA/FOXO3/EGFR/BCL2L1/ITGA6/MTOR | 7 |
| KEGG | NOD-like receptor signaling pathway | 0.26 | 7.14E-04 | CCL2/NAMPT/FADD/BCL2L1/CASP8 | 5 |
| KEGG | TNF signaling pathway | 0.21 | 1.16E-04 | CCL2/FOS/FADD/CASP8 | 4 |
| KEGG | Autophagy signaling pathway | 0.16 | 3.95E-03 | FOS/VEGFA/CCL2 | 3 |
| KEGG | p53 signaling pathway | 0.16 | 3.48E-03 | CDKN2A/BCL2L1/CASP8 | 3 |
| KEGG | Longevity regulating pathway | 0.16 | 4.97E-03 | FOXO3/SIRT1/MTOR | 3 |

Table S5 Associations between CCL2 expression levels and clinicopathological features (chi-squared test).

| Characteristics | CCL2 expression, n (%) | | χ^2^ | *p* value |
| --- | --- | --- | --- | --- |
|  | Low | High |  |  |
| Total | 347 (50.74) | 330 (49.26) |  |  |
| Gender |  |  | 3.146 | 0.076 |
| Male | 231 (34.12) | 198 (29.25) |  |  |
| Female | 116 (17.13) | 132 (19.50) |  |  |
| Age (years) |  |  | 4.360 | 0.037 |
| <= 65 | 175 (25.85) | 140 (20.68) |  |  |
| > 65 | 172 (25.41) | 190 (28.06) |  |  |
| Histology type |  |  | 8.159 | 0.004 |
| LUAD | 147 (21.71) | 176 (26.00) |  |  |
| LUSC | 200 (29.54) | 154 (22.75) |  |  |
| Tumor size |  |  | 6.829 | 0.009 |
| <=5 cm | 282 (41.65) | 292 (43.13) |  |  |
| >5 cm | 65 (9.60) | 38 (5.61) |  |  |
| TNM stage |  |  | 4.029 | 0.045 |
| I-II vs III-IV | 269 (39.73) | 276 (40.77) |  |  |
| TNM stage | 78 (11.52) | 54 (7.98) |  |  |
